# MixBranchNet: a task-adaptive network for glioma segmentation and genotype prediction by exploiting spatial-spectral correlations in CEST MRI

**DOI:** 10.3389/fonc.2026.1770406

**Published:** 2026-04-23

**Authors:** Yanduo Li, Zhekai Chen, Yitian Fan, Yu Meng, Lin Chen

**Affiliations:** Department of Electronic Science, Fujian Provincial Key Laboratory of Plasma and Magnetic Resonance, School of Electronic Science and Engineering, National Model Microelectronics College, Xiamen University, Xiamen, China

**Keywords:** CEST MRI, deep learning, genotype prediction, glioma, MixBranchNet, segmentation, spatial-spectral correlation

## Abstract

**Objective:**

To develop a task-adaptive deep learning-based model, termed MixBranchNet, that leverages spatial-spectral correlations in chemical exchange saturation transfer (CEST) MRI for improved glioma segmentation and genotype prediction.

**Methods:**

MixBranchNet incorporates a dual-branch Mixing Block that extracts spatial and spectral features in parallel through convolutional and self-attention pathways. The model was trained on multi-offset CEST images with 41 frequency offsets from a 3.0 T MRI scanner. Segmentation and genotype prediction tasks were trained separately, with the segmentation output used as the input for the genotype prediction. Manual annotations from two board-certified neuroradiologists were used as the reference standard for segmentation, while genotype labels were obtained from surgical histopathology. Model performance was assessed using the Dice coefficient for segmentation, mean probabilities (P_mean_), accuracy, sensitivity, specificity, F1-score, and AUC for genotype prediction. Five-fold cross-validation was performed within the development cohort using strict patient-level partitioning. A hold-out test set was defined prior to cross-validation and remained fully isolated from training, validation, and model selection procedures.

**Results:**

MixBranchNet achieved a Dice coefficient of 0.84 (95% CIs: 0.80-0.88) for segmentation, outperforming the full Z-spectrum-based fully convolutional network (FCN) (0.61, *p* < 0.001), the MedSAM model based on spatial information (0.80, *p* < 0.001), and the conventional CEST-specific U-Net (0.82, *p* = 0.003). For genotype prediction, MixBranchNet yielded a P_mean_ of 92.66% (95% CIs: 89.44% - 95.27%), and accuracy of 95.00% for IDH mutation, as well as a P_mean_ of 91.07% (95% CIs: 88.98% - 93.37%), and accuracy of 93.11% for MGMT promoter methylation. All results significantly exceeded the performance of conventional CEST quantification techniques and existing deep learning-based models developed for CEST analysis (*p* < 0.05).

**Conclusion:**

MixBranchNet establishes a methodological foundation for spatial-spectral deep learning in CEST MRI and demonstrates encouraging performance for glioma segmentation and genotype prediction within the current single-center cohort.

## 1 Introduction

Glioma is the most common primary malignant brain tumor in adults, representing more than 80% of all malignant intracranial neoplasms (1). Despite therapeutic advances, survival outcomes remain poor, and long-term morbidity among survivors imposes a considerable public health burden (2). Molecular subtypes, such as isocitrate dehydrogenase (IDH) mutations and O6-methylguanine-DNA-methyltransferase (MGMT) promoter methylation, play a critical role in glioma diagnosis, prognosis, and therapeutic response (3). Patients with IDH-mutant gliomas typically exhibit longer overall survival than those with IDH-wildtype tumors (4). Similarly, MGMT promoter methylation enhances sensitivity to temozolomide-based chemoradiotherapy and serves as a useful biomarker for treatment stratification (5). Currently, the gold standard for determining these genotypes relies on histopathological analysis of surgically resected tumor tissue. However, this invasive approach carries inherent risks such as hemorrhage, infection, and sampling bias (6). Therefore, developing noninvasive techniques, particularly MRI-based approaches, for accurate glioma segmentation and genotype prediction is of critical clinical importance.

Chemical exchange saturation transfer (CEST) MRI is a noninvasive molecular imaging technique that detects exchangeable protons by acquiring a series of saturation-weighted images at different frequency offsets (7). For each voxel, the signal intensity across offsets forms a Z-spectrum, which reflects the underlying molecular composition and exchange dynamics. In this framework, the spatial domain of CEST images provides anatomical and morphological information, while the spectral domain (i.e., Z-spectrum) captures metabolite-related molecular characteristics (8, 9). Importantly, these two domains are not independent; instead, CEST data inherently exhibit spatial-spectral correlation, as signal variations across frequency offsets are modulated by tissue-specific properties (10–12). This dual-domain structure offers a comprehensive representation of both morphology and metabolism, providing valuable information for tumor characterization.

Despite the spatial-spectral correlations inherent in CEST MRI, existing approaches for glioma characterization typically process spatial and spectral information separately. Most conventional methods first involve manual or semi-automatic selection of a region of interest (ROI) to delineate tumor tissue, followed by quantification of metabolite-related features within the ROI to predict glioma genotypes, such as amide proton transfer (APT), nuclear Overhauser enhancement (NOE), and semi-solid magnetization transfer (MT) (13–15). Deep learning (DL)-based segmentation networks attempt to automate this process by learning complex spatial features and achieving accuracy comparable to expert delineation. U-Net, for example, captures multiscale spatial context and has demonstrated strong performance even with limited training data, leading to its widespread adoption in medical image segmentation (16–18). More recently, large segmentation models such as the Medical Segment Anything Model (MedSAM) (19), have further advanced the field by enabling prompt-driven tumor localization. DL methods have also been investigated as a substitute for conventional CEST quantification to improve glioma characterization. For instance, the fully connected models that directly ingest the full Z-spectrum can reduce dependence on handcrafted features and avoid the simplifying assumptions inherent to conventional quantification strategies (20, 21). While effective to some extent, each step utilizes only a single dimension of the data, with spatial features used during segmentation and spectral features during quantification. As a result, the intrinsic spatial-spectral correlations embedded in CEST data remain underutilized in glioma characterization, limiting the overall performance and diagnostic potential of current glioma characterization methods.

In this study, we proposed a task-adaptive DL network, termed MixBranchNet, designed to exploit the spatial-spectral correlations in CEST MRI for glioma segmentation and genotype prediction. MixBranchNet incorporates a dual-branch architecture with a Mixing Block that integrates convolutional and self-attention pathways to jointly capture anatomical and metabolite-related features. Comprehensive evaluations demonstrate that MixBranchNet significantly outperforms conventional approaches that process spatial and spectral information separately, showing that the spatial-spectral fusion strategy enhances the accuracy and robustness of glioma characterization.

## 2 Materials and methods

### 2.1 Participants

The study protocol was approved by the local institutional review board, and written informed consent was obtained from all participants. A total of 132 patients who underwent surgical resection and were diagnosed with gliomas based on histopathological examination were included. All tumors were classified according to the 2021 World Health Organization (WHO) criteria (3). Inclusion criteria were: First, completion of both conventional MRI and CEST MRI examinations prior to surgery; Second, no prior biopsy, chemoradiotherapy, or interventional treatment before imaging; Third, availability of IDH mutation status (IDH-mutant or IDH-wildtype) and MGMT promoter methylation status (methylated or unmethylated), determined by next-generation sequencing (NGS) (22). Exclusion criteria included: First, incomplete pathological or molecular data; Second, poor imaging quality due to susceptibility artifacts or motion-related degradation; Third, presence of multifocal, bilateral, or non-mass-like lesions.

### 2.2 MRI acquisition

**Figure 1:**
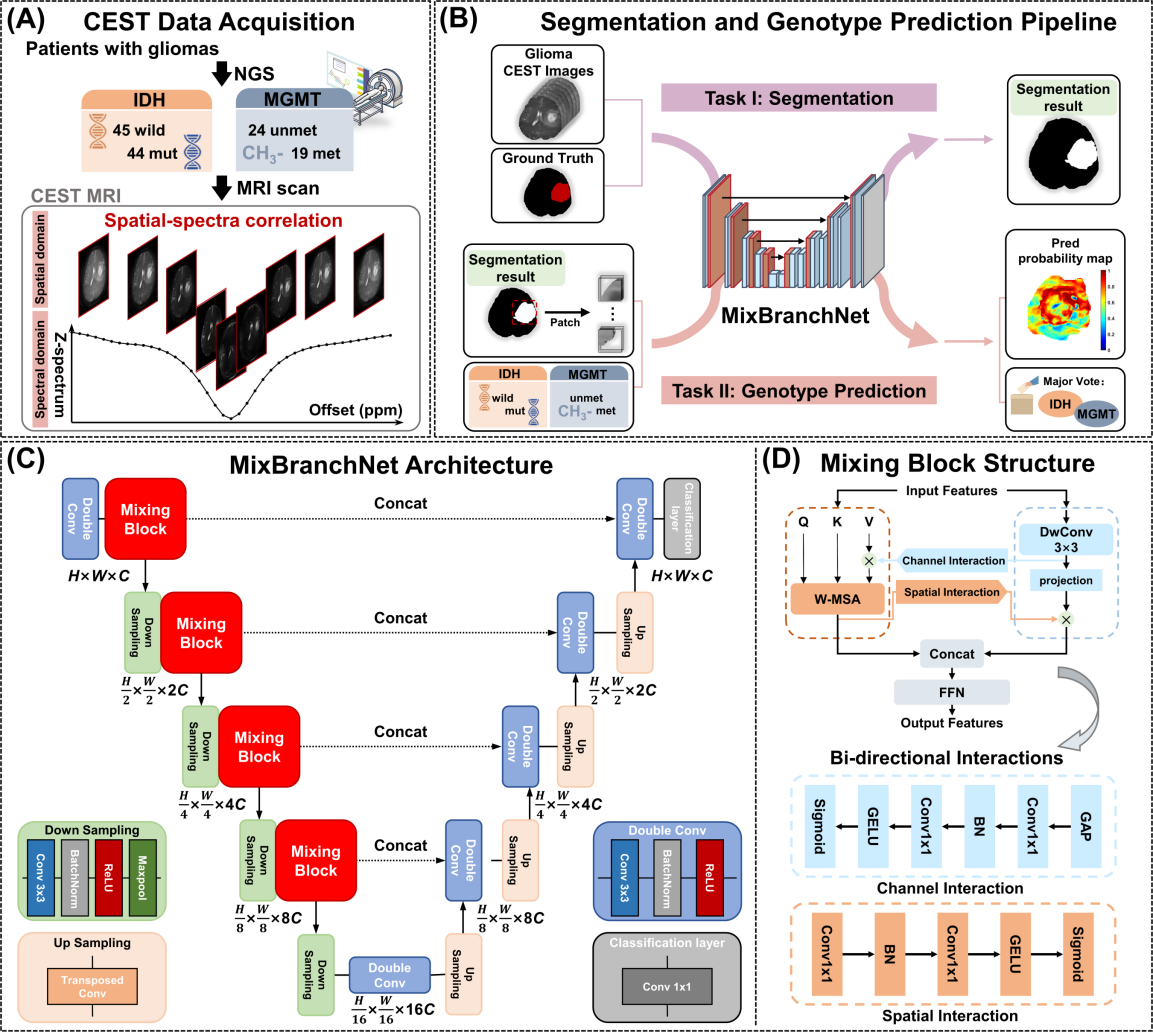
Overview of the proposed framework. **(A)** CEST data acquisition. Multi-offset CEST imaging data is acquired from patients with gliomas, capturing spatial-spectral correlations that vary across different glioma genotypes, such as IDH mutations and MGMT promoter methylation. **(B)** Segmentation and genotype prediction pipeline. The CEST images with ground truth are used to segmentation. Then patches were extracted from segmented region and paired with labels to predict genotypes. Patch-wise predictions are aggregated to form a prediction probability map, and a major vote is applied to obtain the final genotype result. **(C)** MixBranchNet architecture, a U-Net backbone with Mixing Blocks inserted between successive downsampling stages. **(D)** Mixing Block structure, showing the window-based multi-head self-attention (W-MSA) and depthwise convolution (DwConv) branches with bi-directional interactions through a feed-forward network (FFN).

All MRI examinations were performed on a 3.0 T scanner (Ingenia CX, Philips Healthcare, Best, the Netherlands) using a 32-channel phased-array head coil. The imaging protocol included contrast-enhanced T1-weighted (CE-T1W) and fluid-attenuated inversion recovery (FLAIR) sequences for anatomical reference and tumor localization. The CE-T1W sequence was acquired with TR = 7.9 ms, TE = 3.5 ms, field of view (FOV) = 250 × 199 × 170 mm³, and slice thickness = 1 mm. The FLAIR sequence was acquired with TR = 4800 ms, TE = 313 ms, FOV = 250 × 250 × 150 mm³, and slice thickness = 2 mm. CEST MRI was performed using a custom-developed turbo spin-echo (TSE) sequence. The Z-spectrum consisted of one reference image (M_0_, acquired at 100 ppm) and 41 saturation offsets ranging from −5.0 to +5.0 ppm. Radiofrequency (RF) saturation was applied withxa power of 0.9 µT for 3000 ms using continuous wave saturation. This was achieved by utilizing two parallel RF transmission channels operated in a time-interleaved fashion, ensuring a 100% duty cycle saturation. Additional imaging parameters were: TR = 5000 ms, TE = 14 ms, TSE factor = 40, FOV = 200 × 200 mm², matrix size = 80 × 80 (reconstructed to 240 × 240), and voxel size = 2.5 × 2.5 × 4 mm³. The total acquisition time for each CEST scan was 5 minutes and 25 seconds. An overview of the overall workflow, including multi-offset CEST acquisition and spatial-spectral correlations, is illustrated in **Figure 1A**.

### 2.3 Data preprocessing

Structural images (CE-T1W and FLAIR) were resampled and cropped to match the matrix size and field of view of the CEST images. Image registration between CEST and structural images was performed using the Medical Image Registration Toolbox (23) with the M_0_ image as reference. Denoising (10) and B_0_ inhomogeneity correction (24) were applied to improve image quality and ensure voxel-wise consistency of the Z-spectrum. Tumor regions were delineated on co-registered CE-T1W and FLAIR images by two radiologists (3 and 5 years of experience). All preprocessing steps were implemented in MATLAB (R2023b).

### 2.4 Network architecture

The proposed MixBranchNet is built on a modified four-stage U-Net backbone and operates on CEST images of size H × W × C, where H × W are the spatial dimensions and C = 41 denotes the number of Z-spectrum offsets (**Figure  1B**) (16). The encoder performs progressive spatial downsampling with channel expansion (2C, 4C, 8C, 16C), followed by symmetric decoder upsampling, with zero-padding applied to maintain consistency within the resolution hierarchy. Standard convolutional layers between encoding stages were replaced with Mixing Blocks (25). Each Mixing Block integrates local-window multi-head self-attention (W-MSA, 4 × 4 window) (26) and depthwise convolution (3×3 kernel) (27) in parallel. The outputs are concatenated and passed through a feed-forward network (MLP with GELU activation) to enable inter-channel interactions. A total of D = (2, 2, 4, 2) Mixing Blocks were applied across the four stages. To enable inter-branch feature exchange, bi-directional interactions were incorporated, consisting of channel interaction (global average pooling + Conv1×1 + sigmoid) and spatial interaction (channel-reduced Conv1×1 + sigmoid) (**Figure 1C**). The final segmentation or genotype prediction output was generated by a 1 × 1 convolutional layer with softmax activation, producing N = 2 class probability maps for binary tasks.

### 2.5 Model training and pipeline for glioma characterization

The proposed MixBranchNet framework was implemented as a task-adaptive network, in which tumor segmentation and genotype prediction were trained independently but shared the same architecture (**Figure 1D**). For segmentation, glioma regions were delineated by experts to generate binary ground-truth labels, with 0 for normal tissue and 1 for lesion tissue. The preprocessed CEST images and corresponding labels were then used to train the MixBranchNet for tumor segmentation. For genotype prediction, IDH mutation and MGMT promoter methylation statuses determined by next-generation sequencing were assigned as binary labels within the segmented glioma regions (0 = wild-type/unmethylated, 1 = mutant/methylated). To augment training data, CEST images were divided into patches (48 × 48, stride = 12) using a shift-window strategy, and these patches with pixel-wise genotype labels were used to train the MixBranchNet for genotype prediction.

After independent training, the output of the segmentation MixBranchNet was used to extract patches from the tumor regions, which served as input for the genotype prediction MixBranchNet. Patient-level prediction maps were reconstructed by aggregating patches. Finally, the network generated pixel-wise predictions with associated probabilities, which were aggregated to patient-level results using majority voting (threshold = 0.5) and probability mean (P_mean_) (28).

The dataset was randomly divided at the patient level into the development set (85%) for model development and selection, and an independent hold-out test set (15%) for final performance estimation. The hold-out test set remained completely isolated and was not involved in any stage of model training or hyperparameter tuning. Within the development set, internal five-fold cross-validation was conducted to evaluate the generalizability and robustness of the model (29). Specifically, the development set was divided into five approximately equal patient-level subsets. In each fold, four subsets were used for training and one subset was used as a validation set for early stopping and model selection. This process was repeated five times to account for variability across five independent runs. For genotype prediction (IDH and MGMT), stratified sampling was applied during all data splits to preserve class balance. To ensure reproducibility, all random splits were performed using a fixed random seed (seed = 42). The exact number of patients allocated to each fold and to the hold-out test set is provided in **Supplementary Table 4**.

Models were trained using stochastic gradient descent (initial learning rate = 0.0001, momentum = 0.9, weight decay = 1×10^-4^) with batch sizes of 8 for segmentation and 128 for genotype prediction. A weighted combination of binary cross-entropy and Dice loss (30) was used, with class weights of 9.0 for tumor and 1.0 for background to address class imbalance in segmentation. For genotype prediction, class weights were adjusted based on the inverse of class frequencies to mitigate the effects of imbalanced data. To mitigate the risk of overfitting, multiple safeguards were implemented. Model training was monitored using validation set performance, and early stopping was applied if validation Dice loss (segmentation) or validation accuracy (genotype prediction) did not improve for 30 consecutive epochs. This patience threshold was selected based on preliminary experiments showing that validation performance typically stabilized within this range. Training and validation loss curves were continuously monitored to detect potential divergence or instability (**Supplementary Figure 3**). L2 regularization (weight decay, λ = 1×10^-4^) was applied to all trainable parameters. The learning rate was scheduled using a linear warmup followed by cosine annealing: it increased linearly during the first 10 epochs (warmup factor = 0.001) and subsequently decayed according to a cosine schedule (scaling factor = 0.001). Five-fold cross-validation was used to obtain multiple independent estimates of model performance, thereby reducing the influence of any single favorable data split. To examine the effect of training sample size, the model was trained on subsets ranging from 100 to 10,000 samples, and the corresponding training and testing accuracies were recorded. Subsets were generated by randomly sampling patches from each patient, combined with standard data augmentation procedures, including rotation and flipping. All experiments used the same weight initialization protocol to ensure comparability across runs. Training was performed for up to 500 epochs, though early stopping typically terminated training between 150–350 epochs.

Model training was performed on a workstation equipped with an Intel Core i5-13600KF CPU, 32 GB RAM, and an NVIDIA RTX 3090 GPU (24 GB). The implementation was conducted in Python 3.11 using PyTorch 2.0.1.

### 2.6 Patch extraction and aggregation strategy

Patches were extracted from the lesion regions using a shift-window method, as illustrated in **Supplementary Figure 1**. Different patches can be obtained depending on the window size (patch size) and the shift distance (patch stride) for each movement. By adjusting size and stride, we controlled the degree of overlap, as defined in Equation 1:

(1) $$Overlap=(1-\frac{\text{stride}}{\text{size}})\times 100\%$$

Referring to the size of original CEST images, patch sizes of 16, 32, 48, 64, 96, 128, 192 and 240 pixels were tested to determine the most suitable size. A patch size of 240 represents the whole image as input. Moreover, we evaluated the overlaps of 0, 25%, 50%, 75%, and 87.5% by setting different strides under the optimal patch size. The stride is set one-quarter of the patch size by default. The number of patches of different sizes and stride were supplemented in **Supplementary Tables 1, S2**. With the optimal configuration (patch size = 48, stride = 12; 75% overlap), an average of 79 ± 29 patches per patient were extracted (range: 25–162, depending on tumor size). Although patch-based extraction substantially increases the number of training samples, it may introduce spatial correlations among patches derived from the same patient. To prevent pseudo-replication and optimistic bias, all patches from a given patient were assigned to the same data split, ensuring that no patient contributed patches to both the training and evaluation sets. The distribution of patch counts across the five folds is provided in **Supplementary Table 5**.

During patch aggregation, the outer edges of each image patch were cropped so that each pixel was primarily covered by the central region of at least one patch during prediction (31). The crop size was set equal to the stride.

### 2.7 Comparison methods

#### 2.7.1 Segmentation

MixBranchNet was compared with three baselines: a 1D fully convolutional network (FCN-Seg) (32), the prompt-driven MedSAM (19), and a U-Net (17). All models were trained with the same preprocessing, dataset split, losses, optimizer, and learning schedule as MixBranchNet. The FCN-Seg used pixel-wise Z-spectra to generate single-pixel classifications that were assembled into whole-image segmentations. MedSAM, a large pretrained segmentation model; during inference, the M_0_ image and a standard box prompt were provided to guide tumor localization. The U-Net accepted stacked multi-offset CEST images as input channels and produced full-image segmentation masks.

#### 2.7.2 Genotype prediction

MixBranchNet was trained on CEST patches to exploit spatial-spectral correlations and generated patch-wise probabilities that were stitched into patient-level predictions. Its performance was compared with several conventional and DL-based existing methods.

First, conventional CEST quantification techniques, including APTw derived from MTRasym analysis (33) and multi-pool CEST contrasts from Lorentzian fitting (LF) (15), were used to extract spectral information from CEST (details in Supplementary Materials section 2, **Supplementary Table 3**). These quantification results served as input for three models to predict genotypes. Specifically, two logistic regression (LR) models, APTw-LR and LF-LR, were implemented using APTw and LF values, respectively. These models were trained with the fitglm function in MATLAB, utilizing a binomial distribution, logit link function, and parameter optimization via the BFGS algorithm. A convolutional neural network model, APTw-CNN, that utilized APTw for feature extraction (34).

We further compared MixBranchNet with DL networks designed for direct genotype prediction from raw CEST data. A fully connected network (FCN-Pred) used only pixel-wise Z-spectral input to predict genotypes without spatial information (35, 36). The U-Net, similar to MixBranchNet, leveraged spatial-spectral correlations in stacked multi-offset CEST patches. Both models were trained with the same dataset split, optimizer, and learning schedule.

### 2.8 Ablation study

To assess the contribution of spatial-spectral information in segmentation, MixBranchNet was evaluated under two input configurations: (1) the M_0_ image only (“Only-M_0_”) and (2) stacked multi-offset CEST images representing the full Z-spectrum (“Full-CEST”). Both variants used the same network architecture and training settings for fair comparison. To control for channel-number effects between single-channel M_0_ and 41-channel CEST inputs, an additional experiment was conducted in which the M_0_ image was duplicated and stacked to match the channel count of the CEST input (**Supplementary Figure 4**).

To evaluate the contribution of bi-directional interactions between the W-MSA and DwConv branches in MixBranchNet, three ablation experiments were performed on the segmentation and prediction tasks. In experiment 1, only the W-MSA branch was retained; in experiment 2, only the DwConv branch was retained; and in experiment 3, both branches were preserved, but their bi-directional interactions were removed.

### 2.9 Statistical analysis

Segmentation performance was evaluated using the Dice coefficient and intersection over union (IoU). Genotype prediction performance was assessed using the mean prediction probability (P_mean_), accuracy (ACC), sensitivity (SEN), specificity (SPE), F1-score, and area under the ROC curve (AUC). Among these metrics, P_mean_ was selected as the primary evaluation metric because it provides a direct and clinically interpretable measure of patient-level genotype prediction performance. The Pmean was calculated using Equation 2.

(2) $$P_{mean}(C)=\frac{1}{N}\sum_{i=1}^{N}P_{i}(C)$$

where *N* denotes the total number of pixels within the ROI and *P_i_(C)* represents the predicted probability that pixel *i* belonging to genotype class *C*.

Five-fold cross-validation results are reported as mean ± standard deviation (SD) to quantify variability across folds. The coefficient of variation (CoV), defined as the ratio of the SD to the mean of the evaluation metric, was calculated to characterize fold-dependent variability. For the final hold-out test set evaluation, 95% confidence intervals (CIs) were estimated using stratified nonparametric bootstrap resampling at the patient level (B = 2000). Comparisons between MixBranchNet and baseline models were performed using two-sided paired Wilcoxon signed-rank tests at the patient level. The Wilcoxon test was selected because the comparisons were paired within patients and the normality assumptions required for parametric tests could not be reliably justified given the modest sample size. To control for multiple comparisons, the Holm–Bonferroni step-down procedure was applied separately within each task. Adjusted p-values (*p_adj_*) were computed according to the Holm procedure and are reported in **Table 1** and **2**. This procedure controls the family-wise error rate while providing greater statistical power than the classical Bonferroni correction. A threshold of p_adj_ < 0.05 was considered statistically significant. Effect sizes are reported as the mean paired difference (Δ) between MixBranchNet and each baseline model, together with the corresponding 95% bootstrap confidence intervals, to provide interpretable measures of performance improvement. All statistical analyses were performed using MATLAB (R2023b) and SPSS (version 26).

**Table 1:** Segmentation performance on the hold-out test set.

Model	Dice (%)	95% CIs	ΔDice (%)	95% CIs	*p*	*p_adj_*
FCN-Seg	61.01	52.30 – 68.66	23.08	17.47 – 29.43	<0.001	<0.001
MedSAM	79.67	75.49 – 81.95	4.42	3.41 – 7.13	<0.001	<0.001
U-Net	81.71	78.10 – 84.12	2.38	1.76 – 4.09	0.003	0.003
MixBranchNet	**84.09**	**81.12 – 86.75**	–	–	–	–

Values are reported at the patient level on the hold-out test set. The Dice score (Dice) was used as the primary evaluation metric, and ΔDice represents the absolute difference between MixBranchNet and each baseline model. 95% confidence intervals (CIs) for Dice and ΔDice were estimated using patient-level bootstrap resampling (B = 2000). Model comparisons were performed using paired Wilcoxon signed-rank tests, and Holm–Bonferroni correction was applied across the three primary comparisons to obtain adjusted p-values (*p_adj_*). Bold values indicate the best performance in each column.

**Table 2:** Comparison of patient-level genotype prediction performance on the hold-out test set.

Genotype	Method	P_mean_ (%)	95% CIs	ΔP_mean_ (%)	95% CIs	*p*	*p_adj_*
IDH	APTw-LR	64.17	59.57 – 68.47	28.49	23.74 – 33.35	<0.001	<0.001
	LF-LR	73.11	68.14 – 77.48	19.55	15.36 – 24.74	<0.001	<0.001
	APTw-CNN	76.26	70.94 – 80.83	16.40	12.22 – 21.00	<0.001	<0.001
	FCN-Pred	83.53	79.91 – 86.78	9.13	7.14 – 11.56	0.001	0.005
	U-Net	89.04	84.92 – 92.30	3.62	2.45 – 4.83	0.006	0.030
	MixBranchNet	**92.66**	**89.44 – 95.27**	**-**	**-**	**-**	**-**
MGMT	APTw-LR	56.28	49.28 – 62.86	34.79	27.93 – 41.58	0.008	0.039
	LF-LR	63.98	58.62 – 68.69	27.10	22.13 – 32.42	0.008	0.039
	APTw-CNN	72.40	67.14 – 77.33	18.67	14.22 – 23.29	0.008	0.039
	FCN-Pred	79.23	74.23 – 84.85	11.84	8.13 – 15.57	0.008	0.039
	U-Net	86.58	84.05 – 89.34	4.50	2.51 – 6.89	0.039	0.039
	MixBranchNet	**91.07**	**88.98 – 93.37**	**-**	**-**	**-**	**-**

Values are reported at the patient level on the hold-out test set. The mean prediction probability (P_mean_) was used as the primary evaluation metric for genotype prediction. ΔP_mean_ denotes the absolute improvement of MixBranchNet over each baseline method. 95% confidence intervals (CIs) were estimated using patient-level bootstrap resampling (B = 2,000). Model comparisons were performed using paired Wilcoxon signed-rank tests, with Holm–Bonferroni correction applied to adjust the resulting p-values (padj). Bold values indicate the best performance in each column.

## 3 Results

### 3.1 Patient characteristics

Patient demographics and clinical profiles are summarized in **Table 3**. A total of 132 patients with pathologically confirmed gliomas were included in this study, comprising 89 patients for IDH mutation status prediction (50.6% IDH-wildtype, 49.4% IDH-mutant) and 43 patients for MGMT promoter methylation status prediction (55.8% MGMT-unmethylated, 44.2% MGMT-methylated). No significant differences were observed between IDH-wildtype and IDH-mutant groups in gender (*p* = 0.343) or age (*p* = 0.269). However, WHO grade distribution differed significantly (*p* < 0.001): most IDH-wildtype gliomas were grade IV, whereas IDH-mutant gliomas were predominantly grade II. For MGMT promoter status, no significant differences were found between unmethylated and methylated groups in gender (*p* = 0.321), age (*p* = 0.310), or WHO grade (*p* = 0.381).

**Table 3:** Clinical characteristics of the patient population.

Variable	IDH mutation			MGMT methylation			
	Wild type	Mutant	*p*	Unmethylated		Methylated	*p*
No. of patients			NA				NA
	45 (50.6%)	44 (49.4%)		24 (55.8%)	19 (44.2%)		
Age (year)			0.269				0.310
Mean (SD)	50.6 ± 14.2	45.9 ± 11.3		46.7 ± 13.5	45.7 ± 11.2		
Range	20-75	28-69		20-74	28-72		
Gender			0.343				0.321
Male	28	23		15	9		
Female	17	21		9	10		
WHO CNS 5			< 0.001				0.381
Grade IV	30	9		10	6		
Grade III	6	11		5	2		
Grade II	9	24		9	11		

*p* values were calculated using an independent-sample t-test or chi-square test.

IDH, isocitrate dehydrogenase; MGMT, O6-methylguanine-DNA-methyltransferase; SD, standard deviation; WHO CNS5, the fifth edition of the World Health Organization classification of tumors of the central nervous system.

### 3.2 Segmentation performance

In the first stage of the pipeline, the segmentation performance of MixBranchNet was compared with several established baseline models (**Table 4**). FCN-Seg, which relied exclusively on spectral information, achieved Dice = 61.01%, IoU = 47.04%, and ACC = 92.45%. MedSAM, using only spatial information from the M_0_ image, achieved Dice = 79.67%, IoU = 67.59%, and ACC = 95.87%. When the inputs were multi-offset CEST images with spatial-spectral correlations, U-Net achieved higher performance than FCN-Seg and MedSAM (Dice = 81.71%, IoU = 69.67%, and ACC = 97.17%). MixBranchNet outperformed all baseline models, achieving the highest performance with Dice = 84.09%, IoU = 74.16%, and ACC = 97.82%. To provide a more rigorous statistical comparison on the hold-out test set, patient-level statistical inference results are reported in **Table 3**. MixBranchNet achieved a Dice score of 84.09% (95% CI: 81.12% – 86.75%). Compared with FCN-Seg, MedSAM, and U-Net, MixBranchNet demonstrated absolute improvements in Dice of 23.08%, 4.42%, and 2.38%, respectively. All improvements remained statistically significant after correction for multiple comparisons (*p_adj_* < 0.05).

**Table 4:** Cross-validation results of segmentation networks.

Model	Dice (%)	IoU (%)	ACC (%)
FCN-Seg	61.01 ± 1.61	47.04 ± 1.59	92.45 ± 0.69
MedSAM	79.67 ± 1.91	67.59 ± 2.81	95.87 ± 0.54
U-Net	81.71 ± 1.05	69.67 ± 1.13	97.17 ± 0.28
MixBranchNet	**84.09 ± 1.40**	**74.16 ± 1.74**	**97.82 ± 0.20**

Values are presented as mean ± standard deviation. Metrics include Dice score (Dice), intersection over union (IoU), and accuracy (ACC), which together reflect the accuracy and robustness of tumor segmentation based on CEST imaging. Bold values indicate the best performance in each column.

**Figure 2:**
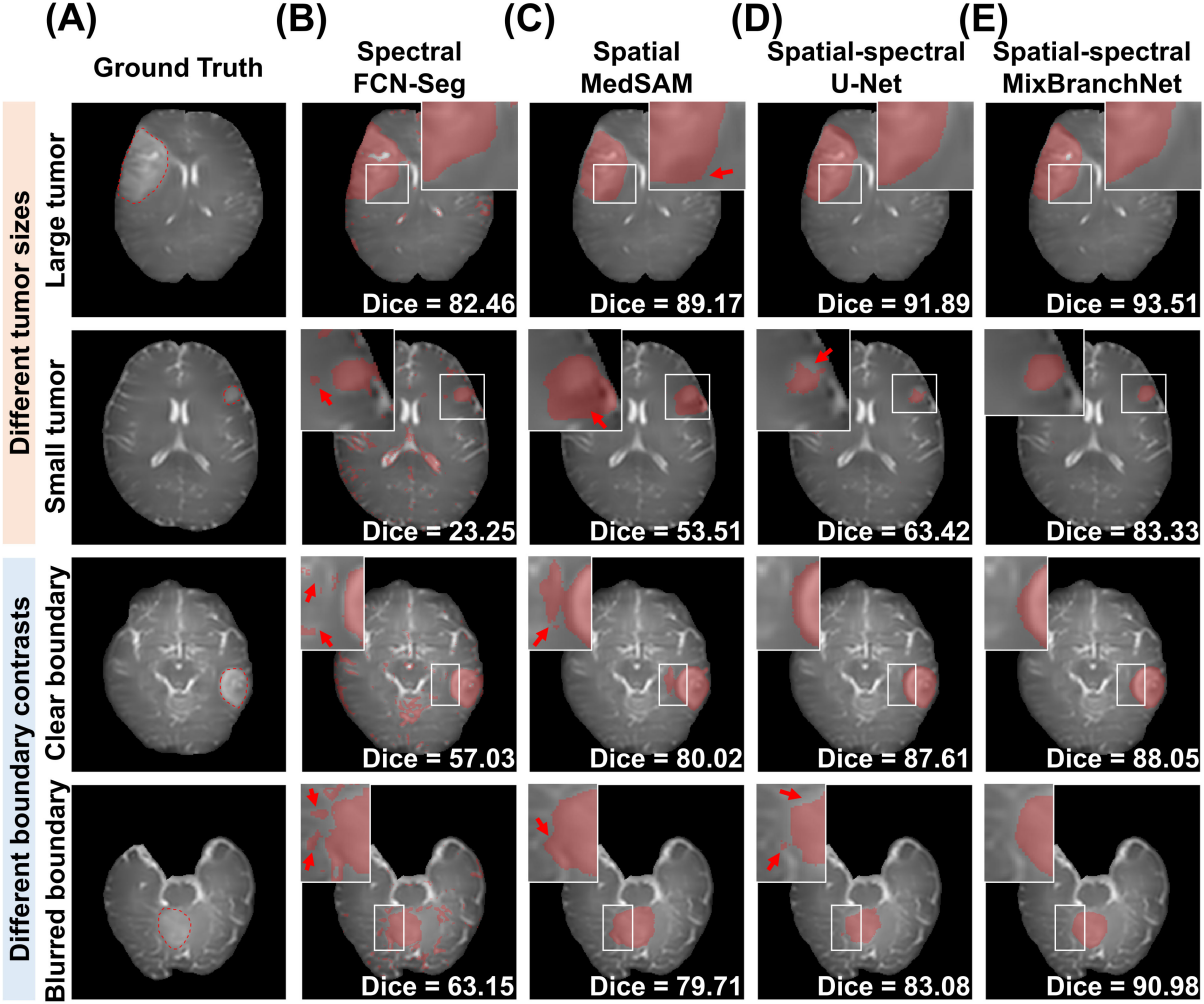
Qualitative comparison of tumor segmentation performance across networks and tumor characteristics. Representative M_0_ images are shown with **(A)** ground truth annotations (red dashed lines) and segmentation results from **(B)** FCN-Seg (spectral information only), **(C)** MedSAM (spatial information only), **(D)** U-Net (spatial-spectral information), and **(E)** MixBranchNet (spatial-spectral correlations). The first two rows illustrate tumors of different sizes (large vs. small), and the last two rows illustrate tumors with different boundary contrasts (clear vs. blurred). Predicted tumor regions are shown as red overlays. Red arrows highlight typical failure regions for each method. Dice scores are reported for all cases and methods.

**Figure 2** presents qualitative and quantitative tumor segmentation results from four representative cases across different lesion sizes (“Small tumor” and “Large tumor”) and boundary conditions (“Clear boundary” and “Blurred boundary”). As expected, all established segmentation methods showed reduced Dice performance in cases with small lesions or blurred lesion boundaries. The FCN-Seg, which relies solely on spectral information, frequently misclassified normal glandular tissue as tumor, producing fragmented and anatomically incoherent outputs with numerous spurious foci and incomplete lesion coverage across all cases (**Figure 2B**). MedSAM generated more coherent and contiguous segmentations due to its strong spatial priors, performing well under high tumor-to-background contrast. However, it frequently exhibited over-segmentation and leakage into surrounding parenchyma in more challenging settings (**Figure 2C**, red arrows), such as low-contrast regions (rows 1 and 4) and in the small-tumor case (row 2). U-Net, with spatial-spectral correlations, performed better than FCN-Seg and MedSAM, but still showed limitations. For instance, the case with small tumor exhibited under-segmentation along the superior margin, while the case with a blurred boundary produced segmentation with boundary distortions and fragmented predictions (**Figure 2D**, red arrows). By contrast, MixBranchNet consistently generated the most accurate and coherent segmentations across all cases (**Figure 2E**, Dice: 93.51%, 83.33%, 88.05%, 90.98%). It exhibited fewer false positives, improved delineation of poorly contrasted lesion margins, and better adherence to true boundaries, confirming the benefit of its spatial-spectral fusion mechanism.

### 3.3 Genotype prediction performance

**Table 2** summarizes the performance metrics for IDH and MGMT genotype prediction, comparing three models based on conventional CEST quantification techniques with three DL-based approaches trained directly on raw CEST data. Among the quantification-based methods, APTw-CNN consistently outperformed the LR baselines (IDH prediction: ACC = 80.91%, AUC = 0.878, and F1-score = 83.30%; MGMT prediction: ACC = 72.79%, AUC = 0.772, and F1-score = 74.55%), while LF-LR achieved higher performance than APTw-LR. DL-based models using raw CEST input provided further improvements, and U-Net generally exceeded the spectrum-only FCN-Pred, highlighting the benefit of exploiting spatial-spectral correlations. MixBranchNet achieved the best overall results for both tasks, reaching ACC = 95.00%, AUC = 0.985, SPE = 96.28%, SEN = 93.73% and F1-score = 94.93% for IDH prediction, and ACC = 93.11%, AUC = 0.969, SPE = 94.14%, SEN = 90.80% and F1-score =94.98% for MGMT prediction. For patient-level statistical inference on the hold-out test set, P_mean_ was used as the primary evaluation metric (**Table 5**). For IDH prediction, MixBranchNet achieved a P_mean_ of 92.66% (95% CI: 89.44% – 95.27%). Compared with all baseline methods, MixBranchNet demonstrated statistically significant improvements (all *p_adj_* < 0.05), with absolute gains (ΔP_mean_) ranging from 3.62% to 28.49%. For MGMT prediction, MixBranchNet achieved a P_mean_ of 91.07% (95% CIs: 88.98% – 93.37%). Improvements over baseline methods were also observed, with ΔP_mean_ ranging from 4.50% to 34.79% (all *p_adj_* < 0.05). In addition, supplementary small-sample robustness analysis results are summarized in **Supplementary Table 9**, where the observed performance advantage of MixBranchNet was further supported by exact paired sign-flip permutation testing and simulation-based power estimation under the current hold-out sample size.

**Table 5:** Cross-validation results of different genotype prediction methods.

Genotype	Method	ACC (%)	AUC (%)	SPE (%)	SEN (%)	F1-score (%)
IDH	APTw-LR	66.42 ± 1.29	70.04 ± 1.13	58.92 ± 1.32	73.43 ± 1.05	70.24 ± 1.09
	LF-LR	79.48 ± 1.16	86.32 ± 0.96	79.86 ± 1.23	78.02 ± 1.25	80.03 ± 0.99
	APTw-CNN	80.91 ± 1.59	87.76 ± 2.16	85.06 ± 4.48	81.02 ± 1.03	83.30 ± 1.39
	FCN-Pred	89.15 ± 0.77	89.34 ± 2.69	91.59 ± 2.39	86.67 ± 0.94	88.80 ± 0.61
	U-Net	90.38 ± 1.26	90.79 ± 0.91	94.46 ± 1.61	86.00 ± 1.26	90.02 ± 1.30
	MixBranchNet	**95.00 ± 0.78**	**98.52 ± 1.68**	**96.28 ± 1.59**	**93.73 ± 2.56**	**94.93 ± 0.86**
MGMT	APTw-LR	53.68 ± 2.36	53.68 ± 2.29	51.45 ± 3.07	55.43 ± 2.04	60.03 ± 2.01
	LF-LR	64.26 ± 2.15	67.43 ± 1.93	68.54 ± 2.83	62.24 ± 2.13	69.01 ± 2.15
	APTw-CNN	72.79 ± 4.19	77.16 ± 6.43	75.41 ± 5.92	67.50 ± 1.80	74.55 ± 2.35
	FCN-Pred	79.43 ± 2.89	87.79 ± 3.38	84.03 ± 4.68	77.39 ± 4.34	83.86 ± 2.61
	U-Net	87.12 ± 3.78	92.15 ± 2.88	88.51 ± 3.86	82.04 ± 1.59	85.02 ± 2.31
	MixBranchNet	**93.11 ± 1.41**	**96.92 ± 1.88**	**94.14 ± 2.60**	**90.80 ± 4.17**	**94.98 ± 1.09**

Values are presented as mean ± standard deviation. The performance metrics include Accuracy (ACC), area under the ROC curve (AUC), specificity (SPE), sensitivity (SEN), and F1-score, which together reflect accuracy and robustness of the IDH and MGMT genotypes.

Bold values indicate the best performance in each column.

**Figure 3:**
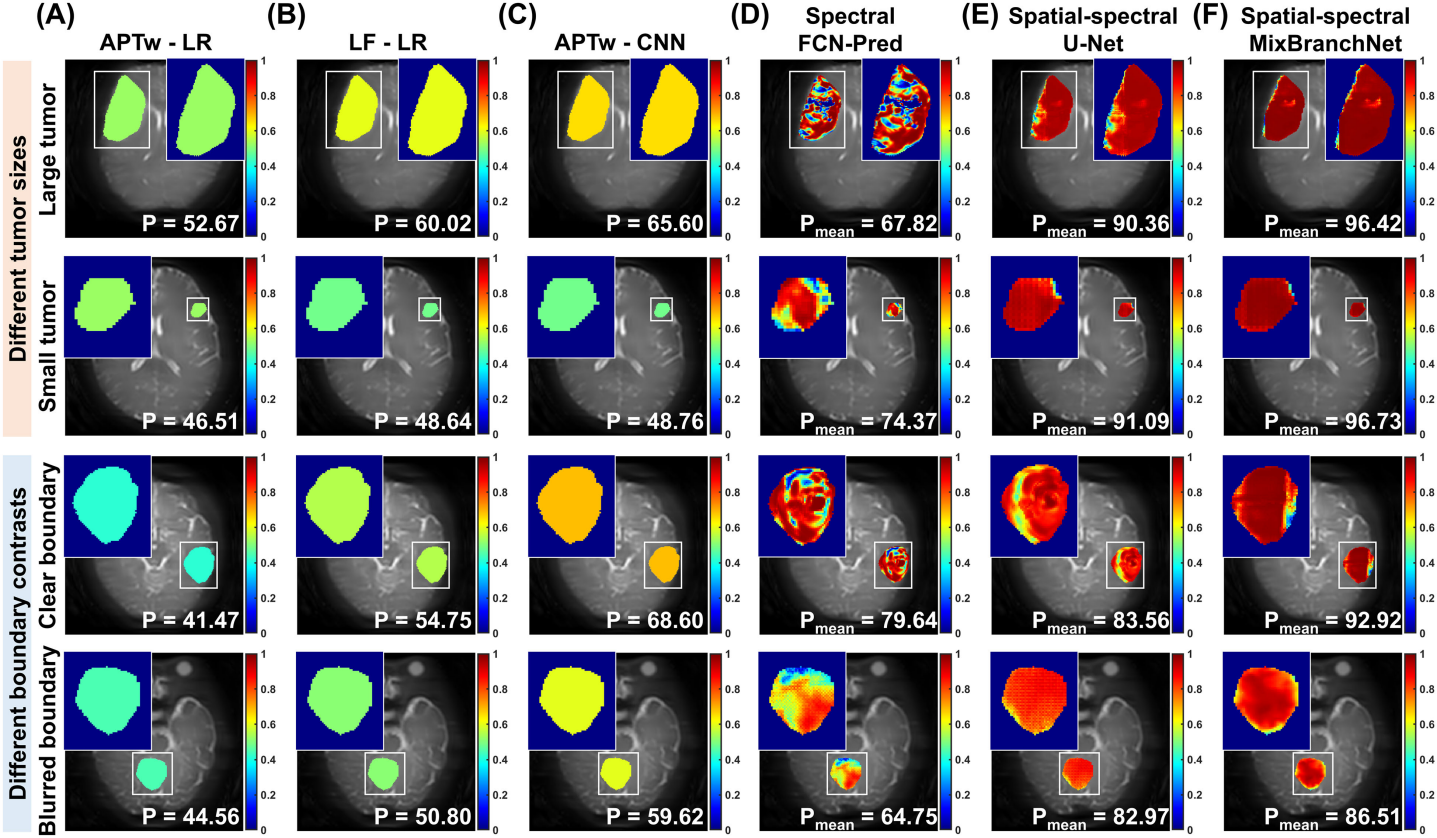
Genotype prediction within segmented tumor regions using different methods and feature types. The first three columns show conventional methods using average APTw signals and Lorentzian fitting parameters, with tumor region colored according to patient-level prediction probabilities. **(A)** APTw-LR and **(B)** LF-LR employ the logistic regression, while **(C)** APTw-CNN uses the convolutional neural network to extract features. The last three columns display pixel-level prediction probability heatmaps generated by **(D)** FCN-Pred, **(E)** U-Net, and **(F)** MixBranchNet, which directly utilize pixel-wise Z-spectra (“Spectral”) and multi-offset CEST images (“Spatial-spectral”) as input. The cases are grouped based on tumor size and boundary contrast. The prediction probability (P) is provided for each method, with P_mean_ representing the mean prediction probability within the tumor region.

**Figure 3** visualizes the genotype prediction probability distributions within the segmented tumor regions, where the tumor masks were obtained from the segmentation results (**Figure 2E**). Conventional quantification-based approaches yield a single probability per case, resulting in uniformly colored tumors and generally modest confidence, as the probabilities remain below 70% across cases (P = 41.47% – 68.60%; **Figures 3A–C**). Specifically, APTw-LR achieved prediction probabilities of 52.67%, 46.51%, 41.47%, and 44.56%, respectively, all lower than the predictions achieved by LF-LR and APTw-CNN. In contrast, DL-based models operating directly on raw CEST data generate pixel-wise probability maps (**Figures 3D–F**). The spectrum-based FCN-Pred achieved higher overall accuracy than three conventional approaches (P_mean_: 67.82%, 74.37%, 79.64%, and 64.75%). This pixel-based model produced errors that lacked morphologic coherence, typically at tumor peripheries and contrast-confounded regions (e.g., along the superior rim or the boundary of small tumors; **Figure 3D**, “Small tumor” and “Blurred boundary”). The U-Net and MixBranchNet, with spatial-spectral correlations, produced more coherent, tumor-constrained probability distributions with substantially higher mean probability across all case types. Notably, MixBranchNet consistently provided the highest performance in each case (P_mean_: 96.42%, 96.73%, 92.92%, and 86.51%), demonstrating robust, high-confidence predictions irrespective of tumor size or boundary definition.

### 3.4 Effect of patch size and overlap

**Figure 4:**
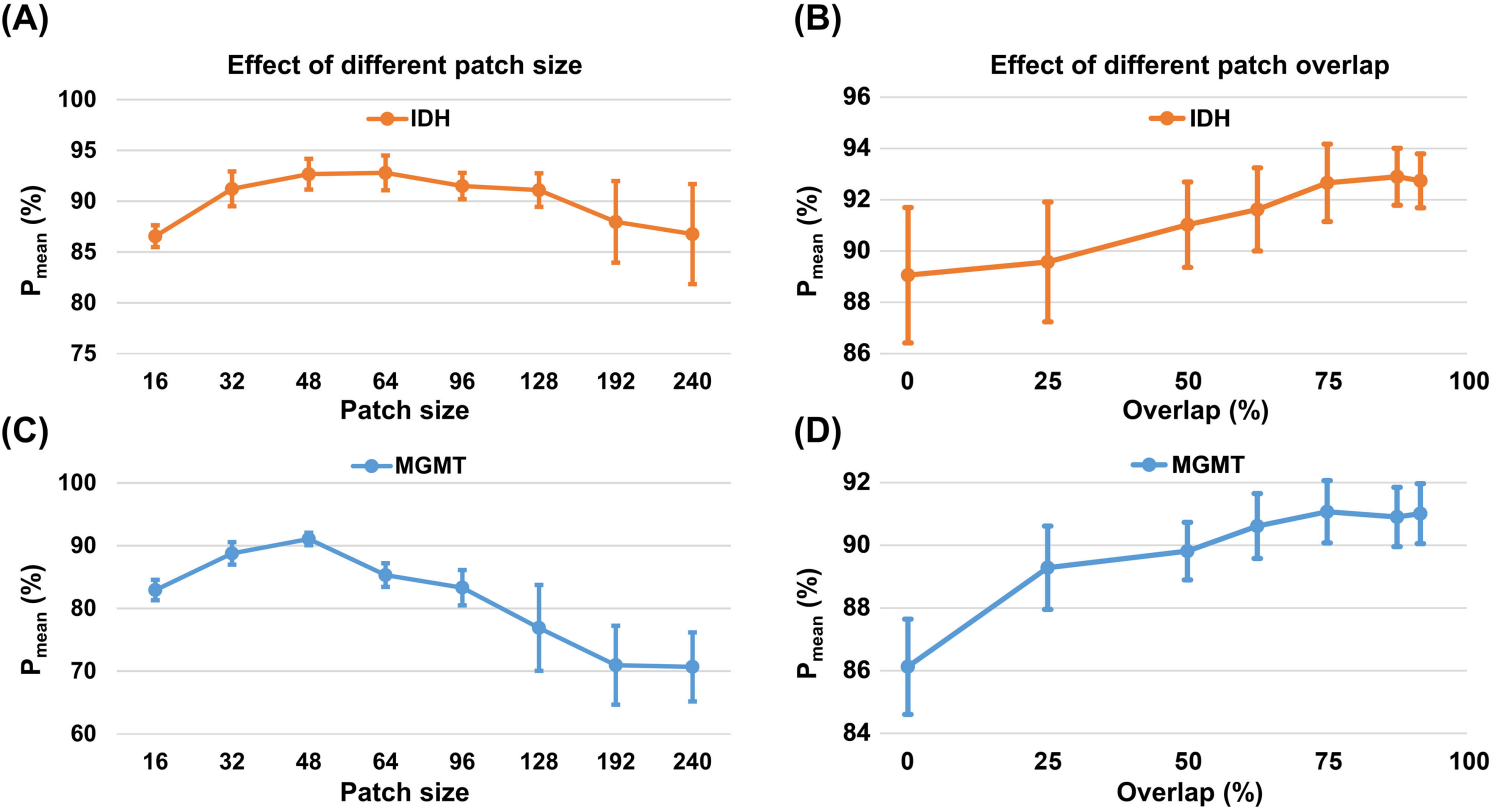
Effect of patch hyperparameter on genotype prediction performance. The line charts present the mean and standard deviation of patient-level prediction probability (P_mean_) across different **(A, C)** patch sizes and **(B, D)** patch overlaps, evaluated via five-fold cross-validation.

**Figure 4** shows the effect of patch size and overlap on genotype prediction accuracy. The genotype prediction performance did not change monotonically with different patch sizes (**Figures 4A, C**). With a patch size of 16, the model showed relatively limited performance (IDH genotype: P_mean_ = 86.56%), remaining close to the pixel-based FCN-Pred (IDH genotype: P_mean_ = 83.53%). When the patch size increased to a specific value, the genotype prediction results reached a peak and then started to decrease when the patch size was larger than this optimal size. The best performance was achieved with patch sizes of 64 × 64 for IDH mutation (P_mean_ = 92.79% ± 1.70%) and 48 × 48 for MGMT promoter methylation (P_mean_ = 91.07% ± 0.99%), sizes that closely approximated the average tumor area in our dataset.

As shown in **Figures 4B, D**, increasing patch overlap improved prediction performance and plateaued at approximately 75% overlap. Beyond this range, further increasing overlap to 87.5% and 91.67% yielded only minimal variation in performance (IDH genotype P_mean_: 92.90% and 92.74%, respectively), while introducing substantial redundancy and higher computational cost. Additional probability maps (**Supplementary Figure 2**) revealed prominent edge effects when patches were extracted without overlap: pixels near patch boundaries received limited spatial context and therefore exhibited reduced predictive accuracy. The degree of overlap was controlled by adjusting the stride (Eq. 1). Based on this analysis, a stride of 12 was selected for the 48 × 48 patch size to achieve the optimal 75% overlap.

### 3.5 Ablation results

**Figure 5:**
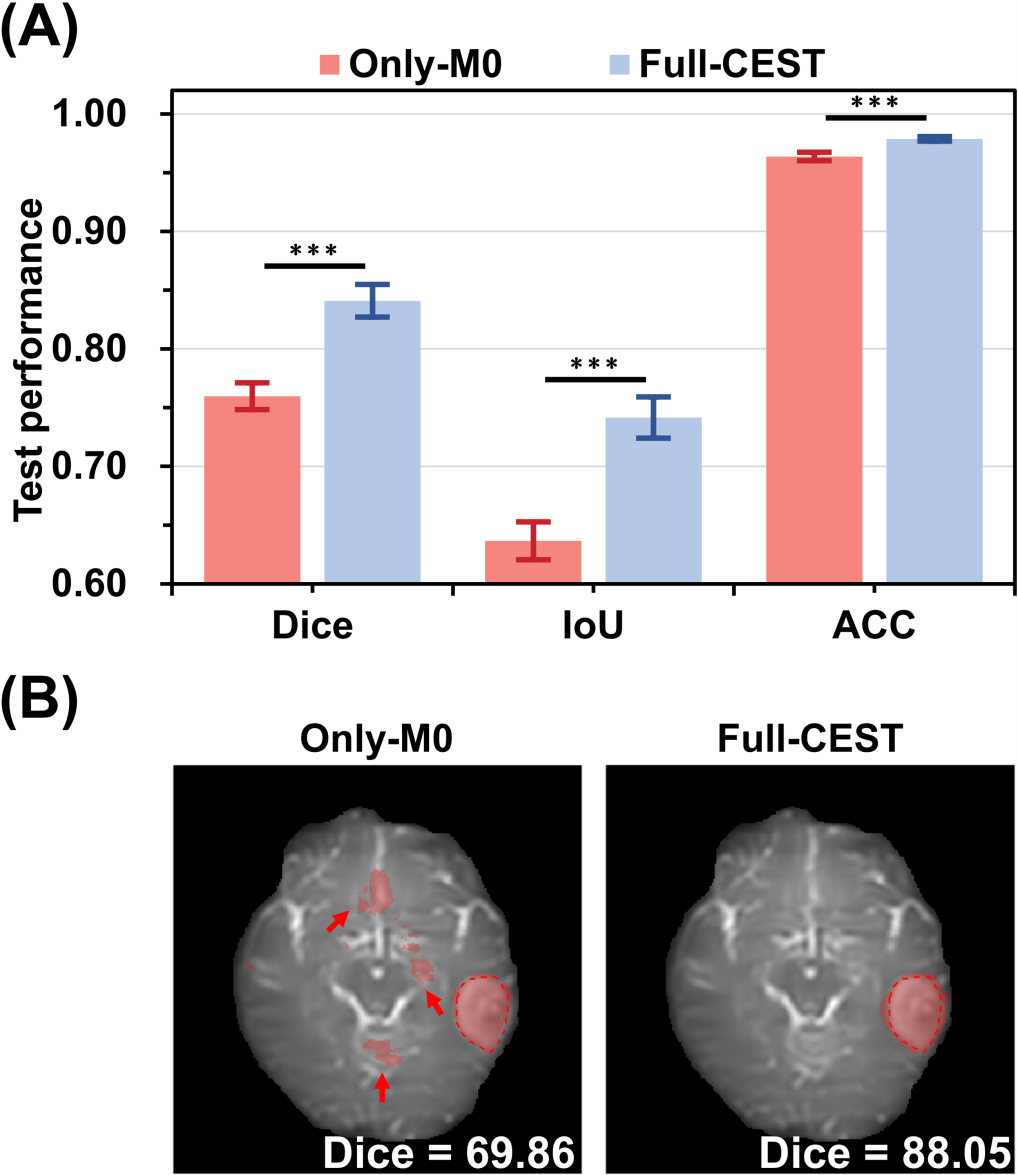
Comparison of M_0_ and multi-offset CEST inputs for glioma segmentation. **(A)** Quantitative evaluation of segmentation performance on the test dataset. **(B)** Representative case showing results from the 100 ppm M_0_ image alone (“Only-M_0_”) versus multi-offset CEST images (−5.0 to +5.0 ppm, “Full-CEST”). Ground truth is outlined on the M_0_ image with red dashed lines, and the segmented tumor regions are overlaid in red. Statistical significance is indicated as follows: *p < 0.05, **p < 0.01, and ***p < 0.001.

As shown in **Figure 5A**, MixBranchNet performance was compared using only the M_0_ image versus multi-offset CEST input with the full Z-spectrum. Segmentation with multi-offset CEST images significantly outperformed the M_0_-only input (*p* ≤ 0.001), achieving Dice = 75.97%, IoU = 63.66%, and ACC = 96.20%. A representative case from the independent hold-out test set further demonstrated improved delineation with multi-offset CEST input, particularly at tumor boundaries with subtle contrast (**Figure 5B**). More experimental results regarding the CEST data input scale were supplemented in **Supplementary Table 7**.

Three ablation experiments were performed to evaluate the contribution of MixBranchNet components (**Table 6**). In experiment 1, only the DwConv branch (“Conv”) was used as a convolution-based U-Net baseline. Incorporating the W-MSA branch (“Attention”) in experiment 2 improved performance, achieving a Dice of 82.56 ± 1.03% for segmentation, F1-score of 92.01 ± 0.77% for IDH genotype prediction, and F1-score of 89.12 ± 1.17% for MGMT genotype prediction, showing improvements of 3.72%, 1.64% and 2.69%, respectively. Adding both branches in parallel in experiment 3 resulted in Dice = 82.95 ± 0.96% for segmentation, F1-score = 92.62 ± 0.70% for IDH prediction, and F1-score = 90.46 ± 1.61% for MGMT prediction, which were similar to experiment 2. In contrast, the full MixBranchNet, which incorporates bi-directional interactions between the convolution and attention branches, achieved the highest performance across all tasks. These findings underscore the importance of interactive fusion between spectral (DwConv) and spatial (W-MSA) processing streams, demonstrating that effective spatial-spectral coupling is critical for both accurate segmentation and robust genotype prediction.

**Table 6:** Ablation study on the model components.

Experiment number	Mixing block			Segmentation dice (%)	IDH prediction F1-score (%)	MGMT prediction F1-score (%)
	onv	Attn	Bi-dir			
1	**✓**			78.84 ± 0.96	90.37 ± 1.26	86.43 ± 2.45
2		**✓**		82.56 ± 1.03	92.01 ± 0.77	89.12 ± 1.17
3	**✓**	**✓**		82.95 ± 0.96	92.62 ± 0.70	90.46 ± 1.61
Ours	**✓**	**✓**	**✓**	**84.09 ± 1.40**	**94.93 ± 0.86**	**94.98 ± 1.09**

Comparison of model variants evaluating the contribution of the depthwise convolution branch (Conv), the local-window self-attention branch (Attn), and bi-directional interactions (Bi-dir). Performance metrics include Dice score of segmentation task, F1-score of IDH prediction task and F1-score of MGMT prediction task. Results are presented as mean ± standard deviation from five-fold cross-validation.

Bold values indicate the best performance in each column.

### 3.6 Robustness of patch-level learning and cross-validation stability

**Figure 6:**
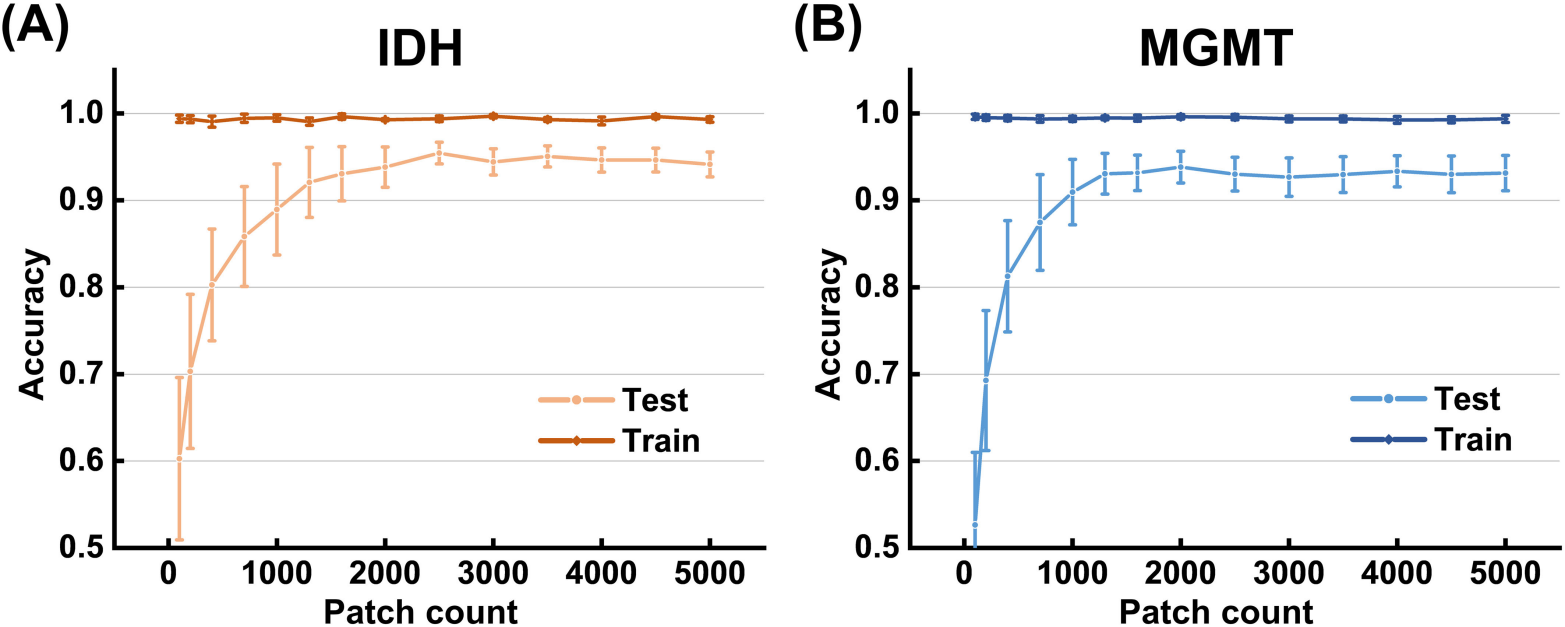
Model performance with different sample sizes. Curves show accuracy for predicting IDH mutation **(A)** and MGMT promoter methylation **(B)** under varying training sample sizes. The curves demonstrate the relationship between the number of training samples and model accuracy, with error bars representing the standard deviation across folds.

To assess the robustness of patch-level training, **Figure 6** illustrates the effect of training data size on MixBranchNet performance for genotype prediction. Test accuracy increased with larger training sets and subsequently reached a plateau. For IDH prediction, ACC increased from 60.27% and stabilized at 95.43% when the training data exceeded 2,500 patches (**Figure 6A**). For MGMT prediction, ACC increased from 53.65% and stabilized at 93.06% at approximately 1,300 patches (**Figure 6B**). In the present study, 75 IDH patients and 35 MGMT patients generated 4,799 and 1,575 training patches, respectively, which was sufficient to achieve stable training performance. In addition, patch-level evaluation produced results highly comparable to patient-level evaluation for both IDH and MGMT prediction, with no significant distributional differences and confirmed statistical equivalence (**Supplementary Figure 5**, **Supplementary Table 8**).

**Table 7** displays the fold-wise test performance of cross-validation. MixBranchNet achieved a mean Dice score of 0.84 for segmentation (range: 0.82–0.86), a mean P_mean_ of 0.93 for IDH prediction (range: 0.91–0.94), and 0.91 for MGMT prediction (range: 0.90–0.92). These results remained consistent across folds, indicating stable model performance. The substantial overlap of 95% CIs further suggests consistent predictive performance and does not indicate meaningful fold-dependent bias. Validation performance also showed low fold-dependent variability, with low CoV across the five folds for all tasks (**Supplementary Table 6**; Segmentation: 5.21%; IDH: 1.57%; MGMT: 3.45%), supporting stable optimization across different data partitions.

**Table 7:** Patient-level test performance across cross-validation folds.

Evaluation metrics	Fold 1 (%)	Fold 2 (%)	Fold 3 (%)	Fold 4 (%)	Fold 5 (%)
Segment-Dice [95% CIs]	86.08 [81.90, 89.89]	83.28 [79.53, 88.03]	84.50 [80.50, 88.87]	82.36 [79.06,87.89]	84.22 [80.46, 88.31]
IDH-P_mean_ [95% CIs]	93.70 [90.21, 96.68]	92.84 [89.59, 96.08]	90.51 [88.76, 95.65]	91.91 [89.06, 95.93]	94.34 [90.68, 97.49]
MGMT-P_mean_ [95% CIs]	90.60 [87.46, 93.02]	90.78 [87.71, 93.73]	91.75 [88.92, 94.03]	89.86 [86.89, 92.62]	92.38 [89.29, 94.74]

Values are presented as means with 95% confidence intervals (CIs). Each column corresponds to one of the five independently trained models obtained from five-fold cross-validation. All metrics were calculated at the patient level. The 95% CIs were estimated using 2,000 paired bootstrap resamples of the hold-out test set.

## 4 Discussion

Accurate glioma segmentation and preoperative genotype prediction are essential for clinical decision-making and prognosis (3). Unified, automated pipelines have been shown to streamline radiological workflows and improve diagnostic consistency (18). In this study, we developed MixBranchNet, a deep learning framework that leverages spatial-spectral correlations in CEST MRI. By jointly integrating spatial and Z-spectral features, MixBranchNet achieved superior performance in both glioma segmentation and genotype prediction compared with existing CEST-based deep learning approaches.

Exploiting spatial-spectral correlations in CEST MRI provides complementary information beyond either domain alone, leading to more accurate glioma segmentation and genotype prediction. For segmentation, most existing methods use spatial features from structural MRI, where differences in morphology and signal intensity delineate tumors based on contrast enhancement, mass effect, and edema (37). Recent studies have shown that the Z-spectrum, which encodes metabolic information, can also be used for tumor segmentation (8). Gliomas are characterized by enhanced glycolysis, accelerated protein turnover, and loss of myelin-associated lipids, leading to distinctive Z-spectral patterns compared with normal tissues (i.e., non-neoplastic gray and white matter). In gray matter, amide proton transfer is lower and lactate effects are minimal, whereas in white matter, strong NOE signals are observed due to abundant myelin (38, 39). These metabolic contrasts enhance tumor-parenchyma separability and support the integration of Z-spectral features into segmentation frameworks. For genotype prediction, most existing approaches rely on Z-spectral features that capture metabolic differences within tumor regions (37). IDH-mutant gliomas, with 2-hydroxyglutarate accumulation and a more alkaline intracellular milieu, typically show significantly higher APT signal intensity compared to IDH-wildtype gliomas, reflecting lactate-driven acidification and accelerated protein turnover (40, 41). NOE signals in IDH-mutant gliomas are generally lower, but may vary, potentially showing weaker intensities compared to IDH-wildtype tumors, which could display higher NOE signal intensities due to increased metabolic activity (14). Similarly, MGMT-unmethylated gliomas sustain high glycolytic flux and oxidative lipid catabolism, resulting in higher APT signal intensities, while MGMT-methylated tumors accumulate lipids and downregulate protein synthesis, producing lower APT signal intensities and potentially higher NOE signal intensities, although direct data on NOE signal strength in relation to MGMT methylation status is limited (33, 42). Additionally, a CEST peak at 2.0 ppm reflects creatine (Cr) metabolism (43), while signals around 1.2 ppm have been linked to glucose−related exchange (44). Moreover, pH changes and semisolid MT effects can also shape the overall Z−spectrum (45). These Z-spectral features capture the complex metabolic and molecular landscape of gliomas, may influence the genotype of glioma. Beyond spectral features, the spatial domain also provides complementary information for genotype prediction. Recent studies have shown that CEST-derived parameters vary across different tumor ROIs, and diagnostic performance depends on the spatial region analyzed (46, 47). Moreover, incorporating spatial context leverages local smoothness to mitigate the susceptibility of Z-spectral quantification to noise and local fluctuations (11, 12), thereby potentially improving the stability and reliability of genotype prediction.

The architectural design of MixBranchNet effectively captures these spatial-spectral dependencies by enabling bi-directional information exchange between convolutional and self-attention pathways. Depthwise convolutions provide channel-specific spectral filtering with local receptive fields (27), whereas window-based self-attention aggregates long-range spatial dependencies while sharing parameters across spectral channels (25). These operations are complementary: the former emphasizes spectral sensitivity but is spatially limited, while the latter captures global spatial context but less spectral detail. By exchanging spatial cues from attention with spectral cues from convolution, the Mixing Block overcomes these limitations and produces fused feature embeddings that jointly encode morphologic and metabolite-related information. This design extends the feature-mixing paradigm of hybrid networks to explicitly model spatial-spectral correlations (48). The bi-directional coupling further enables task adaptability without altering network topology: trained on whole-tumor images, MixBranchNet strengthens spatial context and boundary precision for segmentation, while training on tumor-centered CEST patches highlights discriminative spectral signatures for genotype prediction. In this way, the network accommodates the distinct informational demands of both tasks within a single unified backbone, demonstrating strong task adaptability that not only improves efficiency but also facilitates broader clinical translation by reducing the need for task-specific architectures.

Task-specific input design further enhances performance by balancing the relative importance of spatial and spectral information. For tumor segmentation, whole-image inputs with full Z-spectral information were used, as delineation relies primarily on global morphology and intensity contrasts across the brain, with spectral cues providing complementary support (49). In contrast, genotype prediction is driven predominantly by subtle Z-spectral variations within tumor tissue. Expanding the field of view inevitably includes gray and white matter with strong but noninformative spectra, which can confound and obscure genotype-specific signals (33, 40). Patch-based modeling addresses this challenge by constraining the analysis to tumor-centered regions, thereby exploiting local spatial-spectral correlations while suppressing irrelevant background. In our dataset, the average tumor size was approximately 48 × 48 pixels, and patches of this scale achieved an optimal trade-off between spatial redundancy and spectral stability. Smaller patches underutilized spatial redundancy, while larger patches incorporated more non-tumor tissue, diluting discriminative features and reducing accuracy (50, 51). The boundaries of patches, particularly at their edges, tend to include more non-tumor tissue, which leads to a greater dilution of discriminative features. This dilution makes the prediction of the edge regions less precise, contributing to edge effects. Overlap-based aggregation further smoothed boundaries and reduced edge effects (**Supplementary Figure 2**) (31). An additional comparability analysis between patient-level and patch-level metrics suggests that patch-based learning improves optimization stability without inflating patient-level performance (**Supplementary Table 8**). Thus, segmentation benefits from whole-image inputs where spatial information predominates, whereas genotype prediction benefits from patch-based inputs where spectral features are prioritized and tumor-specific correlations are more effectively captured.

Effective generalization in limited clinical datasets requires careful mitigation of overfitting, and several strategies were adopted in this study to ensure model robustness. In deep learning, the degree of signal separability directly affects the effective sample size and the complexity of learning, with larger margins providing stronger generalization guarantees and reducing sample requirements (52). This explains why segmentation can be reliably trained with fewer cases, as the contrast between tumor and normal tissue is pronounced, whereas genotype prediction requires substantially richer sampling to capture subtle Z-spectral shifts. To mitigate this risk, a patch-based strategy was adopted, which not only concentrates learning on tumor regions but also increases the number of training samples in a label-preserving manner, thereby serving as an effective form of data augmentation (**Supplementary Tables 1, S2**). Beyond this, additional measures were implemented to further reduce overfitting. Models prone to overfitting may appear to perform well on the training set yet fail clinically by memorizing idiosyncratic features and noise (53). To mitigate the risk of overfitting, we adopted recent best-practice recommendations for clinical prediction models (54), including monitoring learning dynamics (**Supplementary Figure 3**), applying early stopping to discourage memorization of noise, and incorporating internal validation across data partitions (**Supplementary Table 6**). Nevertheless, completely ruling out overfitting will ultimately require external validation, and patch-based learning may still introduce optimistic performance estimates because overlapping patches from the same tumor increase optimization instances without a corresponding increase in patient-level diversity. Specifically, higher patch overlap increases the raw number of training patches but does not produce a proportional increase in effective sample size, because neighboring patches share substantial spatial content and become increasingly redundant (55). This may partly account for the performance plateau observed at high overlap ratios (**Figures 4B, D**). To reduce bias related to patch correlation, the final statistical inference was performed at the patient level rather than the patch level. Although such procedures cannot replace external validation, they reduce the likelihood that the observed improvements were driven by sampling noise or unstable optimization. Taken together, these measures support that MixBranchNet learned reproducible spatial-spectral correlations rather than dataset-specific artifacts, underscoring its potential reliability in data-limited clinical imaging scenarios.

MixBranchNet also demonstrates a degree of robustness to acquisition-related perturbations, although this robustness depends on the type and severity of the degradation. The results, shown in **Supplementary Figure 6**, can be observed that MixBranchNet yields comparable performance on corrected data compared to data without B_0_ correction. This suggests that MixBranchNet, with its DL architecture, may possess an inherent robustness to minor distortions introduced by B_0_ inhomogeneity, likely due to the ability to learn spatially localized features and its capacity to adapt to variations in the input data. However, the additional experiment simulating larger B_0_ distortions indicated that B_0_ correction remains beneficial when more severe B_0_ distortions appear (**Supplementary Figure 6B**). In these cases, the distortions might be misinterpreted by the MixBranchNet as part of the relevant frequency offset distribution, leading to incorrect feature learning and a reduced ability to generalize accurately. Consequently, this results in a degradation of performance, as the model begins associating irrelevant distortions with critical clinical features. In addition to B_0_ inhomogeneity, other acquisition-related imperfections, such as reduced signal-to-noise ratio and residual artifacts from motion or inter-offset misalignment, may also degrade the performance of MixBranchNet. To evaluate robustness under degraded acquisition quality, we conducted a test-time Rician noise injection experiment (**Supplementary Figure 7**). Patient-level accuracy showed a monotonic decline with increasing noise levels but remained relatively stable under mild-to-moderate perturbations. Taken together, these findings highlight the importance of robust preprocessing and quality control, including B_0_ correction, image registration, and noise suppression, particularly under challenging acquisition conditions.

It is important to contextualize MixBranchNet within the broader landscape of quantitative imaging approaches for glioma molecular prediction, particularly radiomics. Conventional radiomics typically relies on manually delineated tumor regions and predefined descriptors derived from domain expertise, such as shape, intensity, and texture features, followed by machine learning classifiers for genotype prediction (56). While these approaches offer a degree of interpretability and have demonstrated clinical value, they generally depend on explicit feature engineering and a separate tumor segmentation workflow. In contrast, the proposed framework adopts a data-driven strategy that performs automated tumor segmentation while enabling automatic multi-domain feature extraction. MixBranchNet operates directly on multi-offset CEST images, allowing the network to learn potentially nonlinear patterns without relying on predefined descriptors. Through joint modeling of spatial morphological characteristics and spectral metabolic variations, the network can capture high-dimensional spatial-spectral correlations associated with glioma genotypes that may be difficult to manually specify (48). By reducing reliance on handcrafted features and feature selection procedures, this approach may also mitigate sensitivity to parameter configuration (57). Prior studies have shown that combining complementary imaging modalities can improve glioma characterization and molecular prediction (18, 34). In this context, future extensions of our framework could incorporate additional MRI contrasts, enabling integrated analysis of CEST and anatomical imaging to further explore their complementary contributions.

Translating MixBranchNet into routine clinical practice requires consideration of practical constraints beyond model performance. First, scan time must be compatible with standard clinical workflows. The current protocol requires a relatively long acquisition time (5 min 25 s) for multi-offset CEST imaging. Ongoing technical developments, including accelerated acquisition strategies such as compressed sensing and parallel imaging (58), optimized saturation pulse designs (59), and reduced offset sampling schemes (60), may enable shorter acquisition times (≤ 5 minutes) while maintaining diagnostic utility. Second, standardization of CEST acquisition protocols across scanners and institutions will be essential for ensuring model generalizability (61). Key CEST imaging parameters, including saturation power, saturation duration, frequency offsets, and B_0_/B_1_ field inhomogeneity, vary considerably across research sites (59). Harmonization efforts, such as consensus acquisition recommendations and vendor-supported implementation (21), will therefore be important for future multi-center validation. Our preprocessing pipeline, including B_0_ correction, motion correction, and Z-spectrum normalization, represents an initial step toward standardized image processing, although additional quality control procedures and protocol harmonization will be necessary in future studies. Third, the proposed framework may be extendable to other MRI contrasts (18, 34). The dual-branch architecture of MixBranchNet is conceptually adaptable to other multi-parametric imaging scenarios that integrate spatial structural information with quantitative or spectral imaging features. Overall, this study establishes a methodological foundation for spatial-spectral deep learning in CEST MRI. External validation and protocol harmonization represent logical next steps toward broader clinical adoption.

This study has several limitations. First, the saturation power and duration used in this study (0.9 μT and 3 s) differ from the commonly recommended settings for glioma imaging (2 μT and 2 s) (59). While these parameters enabled effective diagnosis, they may not be optimal. Future work may explore whether optimizing saturation conditions can further improve the performance of the proposed method. Second, genotype prediction relied on pixel-level labels derived from patient-level next-generation gene sequencing outcomes. This could introduce implicit label noise and potential biological inconsistency, because not all tumor regions are guaranteed to share the same molecular phenotype. Prior studies have reported regional molecular heterogeneity in gliomas, including partial cellular expression of IDH mutation in a subset of wild-type gliomas and discordant MGMT promoter methylation across different regions of the same glioblastoma (62, 63). Improving label accuracy by using advanced histological methods that account for tumor heterogeneity may enhance the performance of the proposed method. Third, the modest sample size (89 patients for IDH, 43 for MGMT) limits statistical power and generalizability. While the observed performance advantage was statistically detectable in the current cohort, it remains uncertain whether the corresponding estimates would be equally stable in a larger cohort. Patch-based training increased the number of optimization samples, but did not increase patient-level diversity. Larger independent cohorts will be required to verify the stability of the observed predictive performance, particularly for MGMT prediction. Finally, the proposed framework was evaluated in a single-center cohort under a standardized CEST acquisition and preprocessing pipeline, which is appropriate for proof-of-concept evaluation but may limit external validity (64). Notably, CEST acquisition parameters and processing remain heterogeneous across institutions (59), some degree of performance decline may be anticipated when the model is applied across sites, scanners, and protocols (65). Accordingly, broader clinical translation will require multi-center validation. Cross-center deployment may also benefit from domain adaptation or recalibration strategies to reduce distribution mismatch between training and external data, for example by learning less site-sensitive representations or updating the model with limited target-center data, thereby improving robustness under heterogeneous conditions (66, 67).

## 5 Conclusion

This study demonstrates that MixBranchNet, a task-adaptive deep learning framework, enables accurate glioma segmentation and genotype prediction (IDH and MGMT) by jointly exploiting spatial and spectral features from CEST MRI. CEST MRI provides complementary information for tumor delineation, and spatial-spectral integration improved diagnostic performance compared with existing approaches within the current single-center cohort. These findings suggest that CEST-based deep learning may represent a promising methodological direction for noninvasive glioma characterization, although further validation in larger and multi-center datasets will be required.

## Data Availability

The raw data supporting the conclusions of this article will be made available by the authors, without undue reservation.

## References

[1] Schaff LR Mellinghoff IK . Glioblastoma and other primary brain Malignancies in adults: a review. JAMA. (2023) 329:574–87. doi: 10.1001/jama.2023.0023. PMID: 36809318 PMC11445779

[2] Krishna S Choudhury A Keough MB Seo K Ni L Kakaizada S . Glioblastoma remodelling of human neural circuits decreases survival. Nature. (2023) 617:599–607. doi: 10.1038/s41586-023-06036-1. PMID: 37138086 PMC10191851

[3] Louis DN Perry A Wesseling P Brat DJ Cree IA Figarella-Branger D . The 2021 WHO classification of tumors of the central nervous system: a summary. Neuro Oncol. (2021) 23:1231–51. doi: 10.1093/neuonc/noab106. PMID: 34185076 PMC8328013

[4] Lin MD Tsai ACY Abdullah KG McBrayer SK Shi DD . Treatment of IDH-mutant glioma in the INDIGO era. NPJ Precis Oncol. (2024) 8:149. doi: 10.1038/s41698-024-00646-2. PMID: 39025958 PMC11258219

[5] Kinslow CJ Mercurio A Kumar P Rae AI Siegelin MD Grinband J . Association of MGMT promoter methylation with survival in low-grade and anaplastic gliomas after alkylating chemotherapy. JAMA Oncol. (2023) 9:919–27. doi: 10.1001/jamaoncol.2023.0990. PMID: 37200021 PMC10196932

[6] Weller M Le Rhun E Van den Bent M Chang SM Cloughesy TF Goldbrunner R . Diagnosis and management of complications from the treatment of primary central nervous system tumors in adults. Neuro Oncol. (2023) 25:1200–24. doi: 10.1093/neuonc/noad038. PMID: 36843451 PMC10326495

[7] van Zijl PCM Lam WW Xu J Knutsson L Stanisz GJ . Magnetization transfer contrast and chemical exchange saturation transfer MRI. Features and analysis of the field-dependent saturation spectrum. Neuroimage. (2018) 168:222–41. doi: 10.1016/j.neuroimage.2017.04.045. PMID: 28435103 PMC5650949

[8] Tang PLY Romero AM Nout RA van Rij C Slagter C Swaak-Kragten AT . Amide proton transfer-weighted CEST MRI for radiotherapy target delineation of glioblastoma: a prospective pilot study. Eur Radiol Exp. (2024) 8:123. doi: 10.1186/s41747-024-00523-4. PMID: 39477835 PMC11525355

[9] Ying Y Wang D Zhao Y Quan K Li X Xie Y . The application of quasi-steady-state chemical exchange saturation transfer imaging in the visualization of glioma infiltration and the optimal extent of resection establishment. J Magn Reson Imaging. (2025) 62:480–93. doi: 10.1002/jmri.29786. PMID: 40254958

[10] Chen L Cao S Koehler RC van Zijl PCM Xu J . High-sensitivity CEST mapping using a spatiotemporal correlation-enhanced method. Magn Reson Med. (2020) 84:3342–50. doi: 10.1002/mrm.28380. PMID: 32597519 PMC7722217

[11] Chen X Wu J Yang Y Chen H Zhou Y Lin L . Boosting quantification accuracy of chemical exchange saturation transfer MRI with a spatial-spectral redundancy-based denoising method. NMR BioMed. (2024) 37:e5027. doi: 10.1002/nbm.5027. PMID: 37644611

[12] Chen H Chen X Lin L Cai S Cai C Chen Z . Learned spatiotemporal correlation priors for CEST image denoising using incorporated global-spectral convolution neural network. Magn Reson Med. (2023) 90:2071–88. doi: 10.1002/mrm.29763. PMID: 37332198

[13] Zhu H Li Y Ding Y Liu Y Shen N Xie Y . Multi-pool chemical exchange saturation transfer MRI in glioma grading, molecular subtyping and evaluating tumor proliferation. J Neuro-Oncol. (2024) 169:287–97. doi: 10.1007/s11060-024-04729-9. PMID: 38874844

[14] Zhang X Lu J Liu X Sun P Qin Q Xiang Z . Multipool-CEST and CEST-based pH assessment as predictive tools for glioma grading, IDH mutation, 1p/19q codeletion, and MGMT promoter methylation in gliomas. Front Oncol. (2024) 14:1507335. doi: 10.3389/fonc.2024.1507335. PMID: 39759149 PMC11695364

[15] Su C Xu S Lin D He H Chen Z Damen FC . Multi-parametric Z-spectral MRI may have a good performance for glioma stratification in clinical patients. Eur Radiol. (2022) 32:101–11. doi: 10.1007/s00330-021-08175-3. PMID: 34272981

[16] Ronneberger O Fischer P Brox T . U-Net: Convolutional networks for biomedical image segmentation. In: Proceedings of the medical image computing and computer-assisted intervention (MICCAI), vol. 9351. Cham, Switzerland: Springer. (2015). p. 234–41. doi: 10.1007/978-3-319-24574-4_28

[17] Yang Q Wang M Dou W Ren Y Zhang T Qian L . Parameter map guided explainable segmentation framework for breast cancer using amide proton transfer weighted imaging. Med Phys. (2025) 52:2384–98. doi: 10.1002/mp.17574. PMID: 39699234

[18] Cheng J Liu J Kuang H Wang J . A fully automated multimodal MRI-based multi-task learning for glioma segmentation and IDH genotyping. IEEE Trans Med Imaging. (2022) 41:1520–32. doi: 10.1109/tmi.2022.3142321. PMID: 35020590

[19] Ma J He Y Li F Han L You C Wang B . Segment anything in medical images. Nat Commun. (2024) 15:654. doi: 10.1038/s41467-024-44824-z. PMID: 38253604 PMC10803759

[20] Chen L Schar M Chan KWY Huang J Wei Z Lu H . *In vivo* imaging of phosphocreatine with artificial neural networks. Nat Commun. (2020) 11:1072. doi: 10.1038/s41467-020-14874-0. PMID: 32102999 PMC7044432

[21] Zaiss M Deshmane A Schuppert M Herz K Glang F Ehses P . DeepCEST: 9.4 T Chemical exchange saturation transfer MRI contrast predicted from 3 T data - a proof of concept study. Magn Reson Med. (2019) 81:3901–14. doi: 10.1002/mrm.27690. PMID: 30803000

[22] Zacher A Kaulich K Stepanow S Wolter M Köhrer K Felsberg J . Molecular diagnostics of gliomas using next generation sequencing of a glioma-tailored gene panel. Brain Pathol. (2017) 27:146–59. doi: 10.1111/bpa.12367. PMID: 26919320 PMC8029406

[23] Myronenko A Song X . Intensity-based image registration by minimizing residual complexity. IEEE Trans Med Imaging. (2010) 29:1882–91. doi: 10.1109/TMI.2010.2053043. PMID: 20562036

[24] Yao J Ruan D Raymond C Liau LM Salamon N Pope WB . Improving B0 correction for pH-weighted amine proton Chemical Exchange Saturation Transfer (CEST) Imaging by use of k-means clustering and Lorentzian estimation. Tomography. (2018) 4:123–37. doi: 10.18383/j.tom.2018.00017. PMID: 30320212 PMC6173788

[25] Chen Q Wu Q Wang J Hu Q Hu T Ding E . Mixformer: Mixing features across windows and dimensions. In: Proceedings of the IEEE/CVF conference on computer vision and pattern recognition (CVPR) New Orleans, LA, USA: IEEE. (2022). p. 5249–59. doi: 10.1109/CVPR52688.2022.00518, PMID:

[26] Liu Z Lin Y Cao Y Hu H Wei Y Zhang Z . Swin Transformer: Hierarchical Vision Transformer using shifted windows. In: Proceedings of the IEEE/CVF international conference on computer vision (ICCV) Montreal, QC, Canada: IEEE. (2021). p. 9992–10002. doi: 10.1109/ICCV48922.2021.00986, PMID:

[27] Chollet F . Xception: Deep learning with depthwise separable convolutions. In: Proceedings of the IEEE conference on computer vision and pattern recognition (CVPR) Honolulu, HI, USA: IEEE. (2017). p. 1251–8. doi: 10.1109/CVPR.2017.195, PMID:

[28] Yogananda CGB Wagner BC Truong NCD Holcomb JM Reddy DD Saadat N . MRI-based deep learning method for classification of IDH mutation status. Bioeng Bsl. (2023) 10:1045. doi: 10.3390/bioengineering10091045. PMID: 37760146 PMC10525372

[29] Browne MW . Cross-validation methods. J Math Psychol. (2000) 44:108–32. doi: 10.1006/jmps.1999.1279. PMID: 10733860

[30] Milletari F Navab N Ahmadi S-A . V-Net: Fully convolutional neural networks for volumetric medical image segmentation. In: Proceedings of the 4th International conference on 3D vision (3DV) Stanford, CA, USA: IEEE. (2016). p. 565–71. doi: 10.1109/3dv.2016.79, PMID:

[31] Xu Y Hu S Du Y . Research on optimization scheme for blocking artifacts after patch-based medical image reconstruction. Comput Math Methods Med. (2022) 2022:2177159. doi: 10.1155/2022/2177159. PMID: 35959350 PMC9357777

[32] Zhao Y Chen Y Chen Y Zhang L Wang X He X . A fully convolutional network (FCN) based automated ischemic stroke segment method using chemical exchange saturation transfer imaging. Med Phys. (2022) 49:1635–47. doi: 10.1002/mp.15483. PMID: 35083756

[33] Jiang S Rui Q Wang Y Heo H-Y Zou T Yu H . Discriminating MGMT promoter methylation status in patients with glioblastoma employing amide proton transfer-weighted MRI metrics. Eur Radiol. (2018) 28:2115–23. doi: 10.1007/s00330-017-5182-4. PMID: 29234914 PMC5884703

[34] Yuan Y Yu Y Chang J Chu Y-H Yu W Hsu Y-C . Convolutional neural network to predict IDH mutation status in glioma from chemical exchange saturation transfer imaging at 7 Tesla. Front Oncol. (2023) 13:1134626. doi: 10.3389/fonc.2023.1134626. PMID: 37223677 PMC10200907

[35] Wang S Lu J Zhang X Wang J Chen L . Predicting IDH mutation and MGMT methylation status in glioma patients at the voxel level using CEST-based deep learning. In: Proceedings of the 32nd annual meeting and exhibition of ISMRM Singapore: International Society for Magnetic Resonance in Medicine (ISMRM). (2024). p. Abstract 0402.

[36] Chen Z Lu J Zhang X Wang J Chen L . Enhanced glioma genotype prediction using CEST MRI with full Z-spectrum input, pixel-level learning, and majority voting. NMR BioMed. (2026) 39:e70269. doi: 10.1002/nbm.70269. PMID: 41847841

[37] Jiang S Wen Z Ahn SS Cai K Paech D Eberhart CG . Applications of chemical exchange saturation transfer magnetic resonance imaging in identifying genetic markers in gliomas. NMR BioMed. (2023) 36:e4731. doi: 10.1002/nbm.4731. PMID: 35297117 PMC10557022

[38] Huang J Chen Z Park S-W Lai JHC Chan KWY . Molecular imaging of brain tumors and drug delivery using CEST MRI: promises and challenges. Pharmaceutics. (2022) 14:451. doi: 10.3390/pharmaceutics14020451. PMID: 35214183 PMC8880023

[39] Zhou J Hong X Zhao X Gao JH Yuan J . APT‐weighted and NOE‐weighted image contrasts in glioma with different RF saturation powers based on magnetization transfer ratio asymmetry analyses. Magn Reson Med. (2013) 70:320–7. doi: 10.1002/mrm.24784. PMID: 23661598 PMC3723702

[40] Jiang S Zou T Eberhart CG Villalobos MAV Heo H-Y Zhang Y . Predicting IDH mutation status in grade II gliomas using amide proton transfer-weighted (APTw) MRI. Magn Reson Med. (2017) 78:1100–9. doi: 10.1002/mrm.26820. PMID: 28714279 PMC5561497

[41] Joo B Han K Ahn SS Choi YS Chang JH Kang SG . Amide proton transfer imaging might predict survival and IDH mutation status in high-grade glioma. Eur Radiol. (2019) 29:6643–52. doi: 10.1007/s00330-019-06203-x. PMID: 31175415 PMC6859837

[42] Hao Z Wang J Lv Y Wu W Zhang S Hao S . Identification of MGMT promoter methylation as a specific lipid metabolism biomarker, reveals the feasibility of atorvastatin application in glioblastoma. Metabolism. (2024) 153:155794. doi: 10.1016/j.metabol.2024.155794. PMID: 38301843

[43] Xu J Chung JJ Jin T . Chemical exchange saturation transfer imaging of creatine, phosphocreatine, and protein arginine residue in tissues. NMR BioMed. (2023) 36:e4671. doi: 10.1002/nbm.4671. PMID: 34978371 PMC9250548

[44] Walker-Samuel S Ramasawmy R Torrealdea F Rega M Rajkumar V Johnson SP . *In vivo* imaging of glucose uptake and metabolism in tumors. Nat Med. (2013) 19:1067–72. doi: 10.1038/nm.3252. PMID: 23832090 PMC5275770

[45] Igarashi T Kim H Sun PZ . Detection of tissue pH with quantitative chemical exchange saturation transfer magnetic resonance imaging. NMR BioMed. (2023) 36:e4711. doi: 10.1002/nbm.4711. PMID: 35141979 PMC10249910

[46] Wang X Liu W Masokano IB Liu WV Pei Y Li W . Feasibility of three-dimension Chemical Exchange Saturation Transfer MRI for predicting tumor and node staging in rectal adenocarcinoma: an exploration of optimal ROI measurement. J Imaging Inform Med. (2024) 38:946–56. doi: 10.1007/s10278-024-01029-6. PMID: 39237837 PMC11950466

[47] Chan RW Chen H Myrehaug S Atenafu EG Stanisz GJ Stewart J . Quantitative CEST and MT at 1.5T for monitoring treatment response in glioblastoma: early and late tumor progression during chemoradiation. J Neuro-Oncol. (2021) 151:267–78. doi: 10.1007/s11060-020-03661-y. PMID: 33196965

[48] Kuang H Wang Y Liu J Wang J Cao Q Hu B . Hybrid CNN-transformer network with circular feature interaction for acute ischemic stroke lesion segmentation on non-contrast CT scans. IEEE Trans Med Imaging. (2024) 43:2303–16. doi: 10.1109/TMI.2024.3362879. PMID: 38319756

[49] Zhu Z Wang Z Qi G Mazur N Yang P Liu Y . Brain tumor segmentation in MRI with multi-modality spatial information enhancement and boundary shape correction. Pattern Recognit. (2024) 153:15. doi: 10.1016/j.patcog.2024.110553. PMID: 41936479

[50] Song H Kim Y Kim Y . A patch-based light convolutional neural network for land-cover mapping using landsat-8 images. Remote Sens. (2019) 11:114. doi: 10.3390/rs11020114. PMID: 41725453

[51] Kao PY Shailja S Jiang J Zhang A Khan A Chen JW . Improving patch-based convolutional neural networks for MRI brain tumor segmentation by leveraging location information. Front Neurosci. (2019) 13:1449. doi: 10.3389/fnins.2019.01449. PMID: 32038146 PMC6993565

[52] Lyu S-H Wang L Zhou Z-H . Improving generalization of deep neural networks by leveraging margin distribution. Neural Networks. (2022) 151:48–60. doi: 10.1016/j.neunet.2022.03.019. PMID: 35395512

[53] Zech JR Badgeley MA Liu M Costa AB Titano JJ Oermann EK . Variable generalization performance of a deep learning model to detect pneumonia in chest radiographs: a cross-sectional study. PloS Med. (2018) 15:e1002683. doi: 10.1371/journal.pmed.1002683. PMID: 30399157 PMC6219764

[54] Efthimiou O Seo M Chalkou K Debray T Egger M Salanti G . Developing clinical prediction models: a step-by-step guide. BMJ. (2024) 386:e078276. doi: 10.1136/bmj-2023-078276. PMID: 39227063 PMC11369751

[55] Rutterford C Copas A Eldridge S . Methods for sample size determination in cluster randomized trials. Int J Epidemiol. (2015) 44:1051–67. doi: 10.1093/ije/dyv113. PMID: 26174515 PMC4521133

[56] Riberdy V Guida A Rioux J Brewer K . Radiomics in preclinical imaging research: methods, challenges and opportunities. NPJ Imaging. (2025) 3(1):45. doi: 10.1038/s44303-025-00104-z. PMID: 40983685 PMC12454655

[57] LeCun Y Bengio Y Hinton G . Deep learning. Nature. (2015) 521:436–44. doi: 10.1038/nature14539. PMID: 26017442

[58] She H Greer JS Zhang S Li B Keupp J Madhuranthakam AJ . Accelerating chemical exchange saturation transfer MRI with parallel blind compressed sensing. Magn Reson Med. (2019) 81:504–13. doi: 10.1002/mrm.27400. PMID: 30146714 PMC6497066

[59] Zhou J Zaiss M Knutsson L Sun PZ Ahn SS Aime S . Review and consensus recommendations on clinical APT‐weighted imaging approaches at 3T: Application to brain tumors. Magn Reson Med. (2022) 88:546–74. doi: 10.1002/mrm.29241. PMID: 35452155 PMC9321891

[60] Cheema K Han P Lee HL Xie Y Christodoulou AG Li D . Accelerated CEST imaging through deep learning quantification from reduced frequency offsets. Magn Reson Med. (2024) 93:301–10. doi: 10.1002/mrm.30269. PMID: 39270056 PMC13317472

[61] Perlman O Zhu B Zaiss M Rosen MS Farrar CT . An end-to-end AI-based framework for automated discovery of rapid CEST/MT MRI acquisition protocols and molecular parameter quantification (AutoCEST). Magn Reson Med. (2022) 87:2792–810. doi: 10.1002/mrm.29173. PMID: 35092076 PMC9305180

[62] Preusser M Wöhrer A Stary S Höftberger R Streubel B Hainfellner JA . Value and limitations of immunohistochemistry and gene sequencing for detection of the IDH1-R132H mutation in diffuse glioma biopsy specimens. J Neuropathol Exp Neurol. (2011) 70:715–23. doi: 10.1097/NEN.0b013e31822713f0. PMID: 21760534

[63] Gempt J Withake F Aftahy AK Meyer HS Barz M Delbridge C . Methylation subgroup and molecular heterogeneity is a hallmark of glioblastoma: implications for biopsy targeting, classification and therapy. ESMO Open. (2022) 7:100566. doi: 10.1016/j.esmoop.2022.100566. PMID: 36055049 PMC9588899

[64] Collins GS de Groot JA Dutton S Omar O Shanyinde M Tajar A . External validation of multivariable prediction models: a systematic review of methodological conduct and reporting. BMC Med Res Methodol. (2014) 14:40. doi: 10.1186/1471-2288-14-40. PMID: 24645774 PMC3999945

[65] Yu AC Mohajer B Eng J . External validation of deep learning algorithms for radiologic diagnosis: a systematic review. Radiol Artif Intell. (2022) 4:e210064. doi: 10.1148/ryai.210064. PMID: 35652114 PMC9152694

[66] Guan H Liu M . Domain adaptation for medical image analysis: a survey. IEEE Trans BioMed Eng. (2022) 69:1173–85. doi: 10.1109/tbme.2021.3117407. PMID: 34606445 PMC9011180

[67] Baldeon-Calisto M Lai-Yuen SK Puente-Mejia B . StAC-DA: Structure aware cross-modality domain adaptation framework with image and feature-level adaptation for medical image segmentation. Dig Health. (2024) 10:20552076241277440. doi: 10.1177/20552076241277440. PMID: 39229464 PMC11369866

