# Supplementary material for: MixBranchNet: a task-adaptive network for glioma segmentation and genotype prediction by exploiting spatial-spectral correlations in CEST MRI
Source: Front Oncol. 2026 Apr 23;16:1770406. doi: 10.3389/fonc.2026.1770406 (PMC13149081; doi:10.3389/fonc.2026.1770406)
Supplement: Supplementary file 1 [file DataSheet1.docx]

Supplementary Material

# Section 1. Patch strategy for data augmentation.

The patch strategy was proposed for data augmentation. Additionally, we extracted CEST image patches from local spatial neighborhoods across the entire Z-spectral bands as inputs to deep learning networks to learn spatial-spectral correlations.

## Shift-window patch strategy

Patches were extracted from the lesion regions using a shift-window method, as illustrated in **Figure S1**. First, lesions were cropped from the original images according to the rectangular bounding boxes. Then a square window was shifted from the top-left to the bottom-right of the image, row by row, and pixels within the window formed a patch.


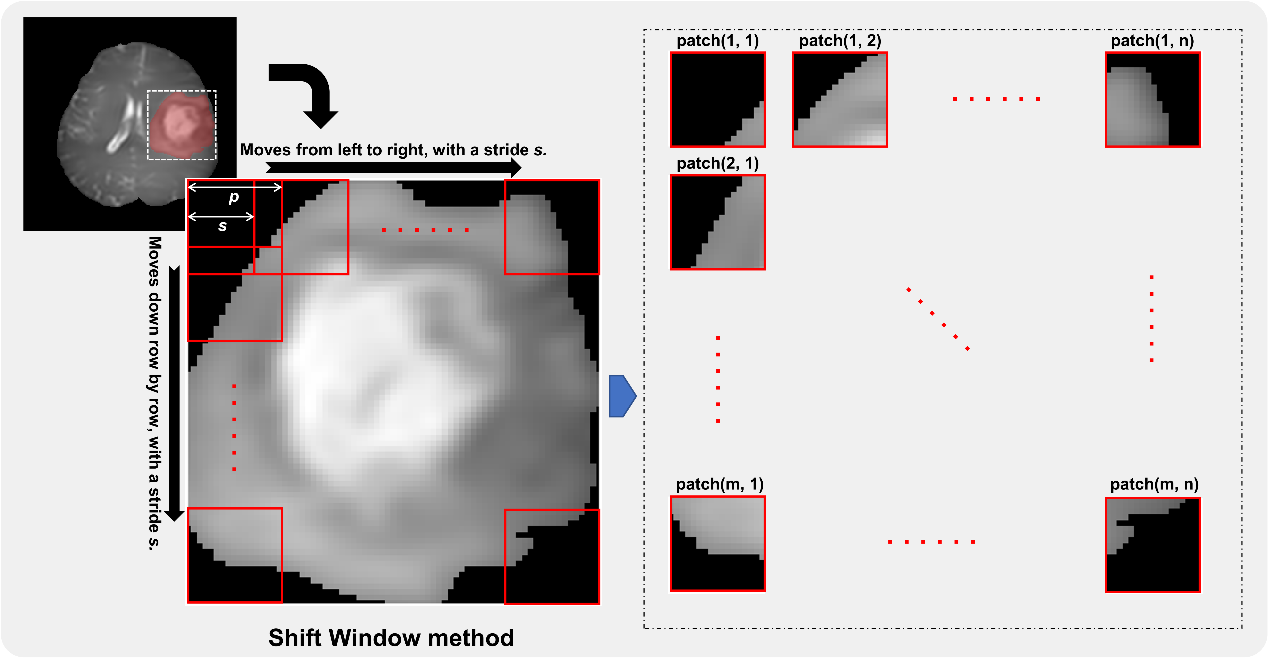


**Supplementary Figure S1.** Shift-window patch strategy. Patches are extracted using a square window with size *p*, and the window is shifted by a distance of stride *s* each time.

## The hyperparameter of patch strategy

Patch size was an important hyperparameter in our method. As shown in **Table S1**, it controlled both the area and the number of patches. As the patch size increased, each patch contained more areas, and the number of patches that could be obtained from the whole CEST image decreased dramatically. Patch overlap, controlled by the patch stride, was another key hyperparameter. As shown in **Table S2**, overlapping patches could also provide a certain degree of data augmentation. Moreover, it provided an opportunity for the pixels at the patch edges to be positioned in the central area of another patch. The pixel-level predicted classes and probability distribution map with different overlaps were shown in **Figure S2**.

**Supplementary Table S1.** The number of training patches with different patch sizes.

| **Patch size (p × p)** | **Number of patches** | | | | | |
| --- | --- | --- | --- | --- | --- | --- |
|  | **IDH_wild_** | **IDH_mut_** | **All** | **MGMT_unmet_** | **MGMT_met_** | **All** |
| 16 × 16 | 10678 | 10427 | 21105 | 3713 | 3319 | 7032 |
| 32 × 32 | 4033 | 3961 | 7994 | 1474 | 1183 | 2657 |
| 48 × 48 | 2423 | 2376 | 4799 | 836 | 739 | 1575 |
| 64 × 64 | 1605 | 1567 | 3172 | 569 | 463 | 1032 |
| 96 × 96 | 755 | 737 | 1492 | 268 | 221 | 489 |
| 128 × 128 | 426 | 414 | 840 | 153 | 123 | 276 |
| 192 × 192 | 38 | 37 | 75 | 20 | 15 | 35 |
| 240 × 240 | 38 | 37 | 75 | 20 | 15 | 35 |

Patches are extracted using a square window of size *p*, and the window is shifted by a distance of stride *s* each time. IDH_wild_ = IDH wild type; IDH_mut_ = IDH mutation; MGMT_unmet_ = MGMT unmethylated; MGMT_met_ = MGMT methylated.

**Supplementary Table S2.** The number of training patches with different patch overlaps.

| **Patch**  **overlap (%)** | **Patch**  **stride** | **Patch number** | | |
| --- | --- | --- | --- | --- |
|  |  | **IDH** | **MGMT** | **All** |
| 0 | 48 | 229 | 97 | 326 |
| 25 | 36 | 385 | 171 | 556 |
| 50 | 24 | 866 | 392 | 1258 |
| 62.5 | 18 | 1475 | 698 | 2173 |
| 75 | 12 | 3172 | 1575 | 4747 |
| 87.5 | 6 | 12691 | 6302 | 18993 |
| 91.67 | 4 | 28563 | 14134 | 42697 |


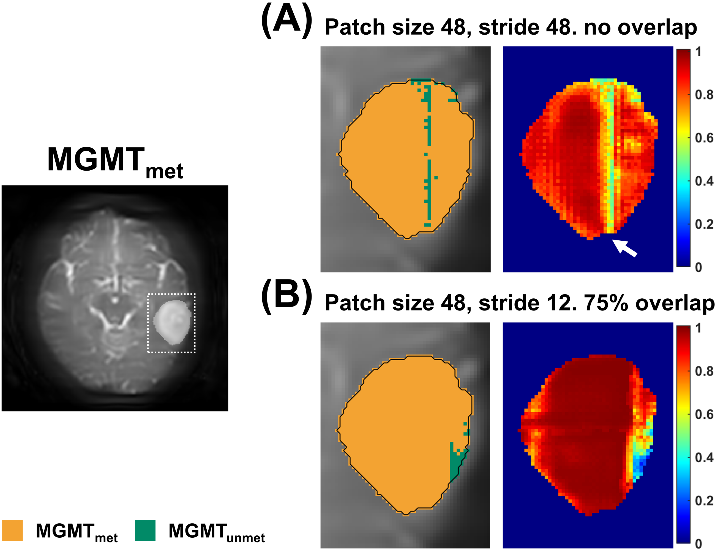


**Supplementary Figure S2.** Patient-level predicted class and probability distribution maps with different overlaps. For predicted class maps, a probability threshold of 0.5 was used to classify each pixel prediction. The class predicted by each pixel is marked by color (MGMT_met_, yellow; MGMT_unmet_, green). Block artifacts are visible in the probability maps (white arrows).

# Section 2. The Traditional CEST Quantification Techniques for Comparison.

## APTw derived from MTR_asym_ analysis

For MTR_asym_ analysis, the quantification result is obtained by calculating the difference in normalized signal intensity at symmetrical frequencies ($\pm\Delta\omega$) relative to the water resonance frequency in the Z-spectrum, defined by:

$MTR_{asym}(\Delta\omega)=\frac{S_{sat}(-\Delta\omega)-S_{sat}(\Delta\omega)}{S_{0}}$ (S1)

where $S_{sat}\left(-\Delta\omega\right)$ and $S_{sat}\left(\Delta\omega\right)$ are the water signal intensities after saturation with radiofrequency irradiation at negative and positive frequency offset ($\Delta\omega$) relative to water, and $S_{0}$ is the image without radiofrequency saturation. The APTw contrast was obtained by setting $\Delta\omega$ to 3.5ppm.

## Multi-pool Lorentzian fitting

Multi-pool Lorentzian fitting is used to extract and quantify CEST contrasts. It assumes a Lorentzian lineshape for each CEST pool, and the Z-spectrum is a combination of these lineshapes, which is described as follows:

$Z= \sum_{i=1}^{N} \frac{A_{i}}{1+4\left(\frac{\Delta\omega-\delta_{i}}{\sigma_{i}}\right)^{2}}$ (S2)

where $\Delta\omega$ is the frequency offset from the water resonance, $A_{i}$ , $\delta_{i}$, and $\sigma_{i}$ denote the amplitude, chemical shift, and full-width half maximum (FWHM) of the ith pool, respectively. In this study, five pools were applied, including direct saturation of water (DS), amide proton transfer effect (APT), nuclear Overhauser effect (NOE), semi-solid magnetization transfer (MT), and the 2-ppm CEST peak (CEST@2ppm). The parameter settings are detailed in **Table S3**.

**Supplementary Table S3.** Multi-pool Lorentzian fitting parameter configuration.

|  |  | **DS** | **APT** | **NOE** | **MT** | **CEST@2ppm** |
| --- | --- | --- | --- | --- | --- | --- |
| **Amplitude** | up | 1 | 0.2 | 0.2 | 0.0025 | 0 |
|  | iv | 0.7 | 0.1 | 0.1 | 0.2 | 0.12 |
|  | lb | 0.4 | 0 | 0 | 0.3 | 1.2 |
| **FWHM** | up | 5 | 10 | 12.5 | 10 | 1.115 |
|  | iv | 2.5 | 5 | 4 | 20 | 2.23 |
|  | lb | 1 | 2.5 | 2 | 100 | 11.15 |
| **Frequency offset** | up | 1 | 3.3 | -3.3 | -1.5 | 2 |
|  | iv | 0 | 3.5 | -3.5 | -1.5 | 2 |
|  | lb | -1 | 3.7 | -3.7 | -1.5 | 2 |

# Section 3. Patient-Level Data Splitting

To ensure transparency and allow independent evaluation of potential bias, we provide a detailed summary of the patient-level data partitioning. The detailed patient distribution is provided in **Table S4**. For each task, approximately 15% of patients (rounded up to the nearest integer) were randomly assigned to an independent hold-out test set, and the remaining 85% constituted the development set. Five-fold cross-validation was conducted within the development set. In each fold, four subsets were used for training and one subset for validation (early stopping and model selection). **Table S5** shows the patch counts distribution across all five cross-validation folds for both IDH and MGMT prediction tasks.

**Supplementary Table S4.** Patient-level data distribution across five-fold cross-validation and the hold-out test set.

| **Number of patients** | **Segmentation** | **IDH** | | **MGMT** | |
| --- | --- | --- | --- | --- | --- |
|  |  | **Wild type** | **Mutant** | **Unmethylated** | **Methylated** |
| **Validation set** | | | | | |
| Fold 1 | 22 | 8 | 8 | 4 | 3 |
| Fold 2 | 22 | 8 | 8 | 4 | 3 |
| Fold 3 | 22 | 8 | 7 | 4 | 3 |
| Fold 4 | 22 | 7 | 7 | 4 | 3 |
| Fold 5 | 22 | 7 | 7 | 4 | 3 |
| **Test set** | | | | | |
| Test set | 22 | 7 | 7 | 4 | 4 |

**Supplementary Table S5.** Patch-level class distribution across cross-validation folds.

| **Number of patches** | **IDH** | | **MGMT** | |
| --- | --- | --- | --- | --- |
|  | **Wild type** | **Mutant** | **Unmethylated** | **Methylated** |
| Fold 1 | 529 (52.1%) | 487 (47.9%) | 151 (54.7%) | 125 (45.3%) |
| Fold 2 | 669 (56.5%) | 515 (43.5%) | 201 (56.0%) | 158 (44.0%) |
| Fold 3 | 523 (53.9%) | 448 (46.1%) | 233 (52.4%) | 212 (47.6%) |
| Fold 4 | 355 (47.0%) | 401 (53.0%) | 149 (47.9%) | 162 (52.1%) |
| Fold 5 | 417 (47.8%) | 455 (52.2%) | 102 (55.4%) | 82 (44.6%) |
| All | 2493 (51.9%) | 2306 (48.1%) | 836 (53.1%) | 739 (46.9%) |

# Section 4. Training accuracy and convergence information of the network.

## Learning-curve analysis

**Figure S3** shows the learning curves for the MixBranchNet on the three tasks: glioma lesion segmentation, IDH genotype prediction, and MGMT genotype prediction. Each panel in **Figure S3** contains two plots: the training and validation loss curves (upper subplot) and the corresponding performance metric curves (lower subplot, Dice coefficient or accuracy). Over the course of 500 epochs, all tasks exhibited steadily decreasing training loss, accompanied by increasing Dice or accuracy that eventually plateaued. The validation metrics remained stable and closely tracked the training metrics throughout training. Notably, a slight increase in validation loss was observed after epoch 400 in segmentation task and around epoch 490 in MGMT prediction task, indicating a potential risk of overfitting in the later stages of training. To mitigate overfitting, an early stopping strategy was employed. Training information was ignored if the validation loss did not decrease for 30 consecutive epochs.


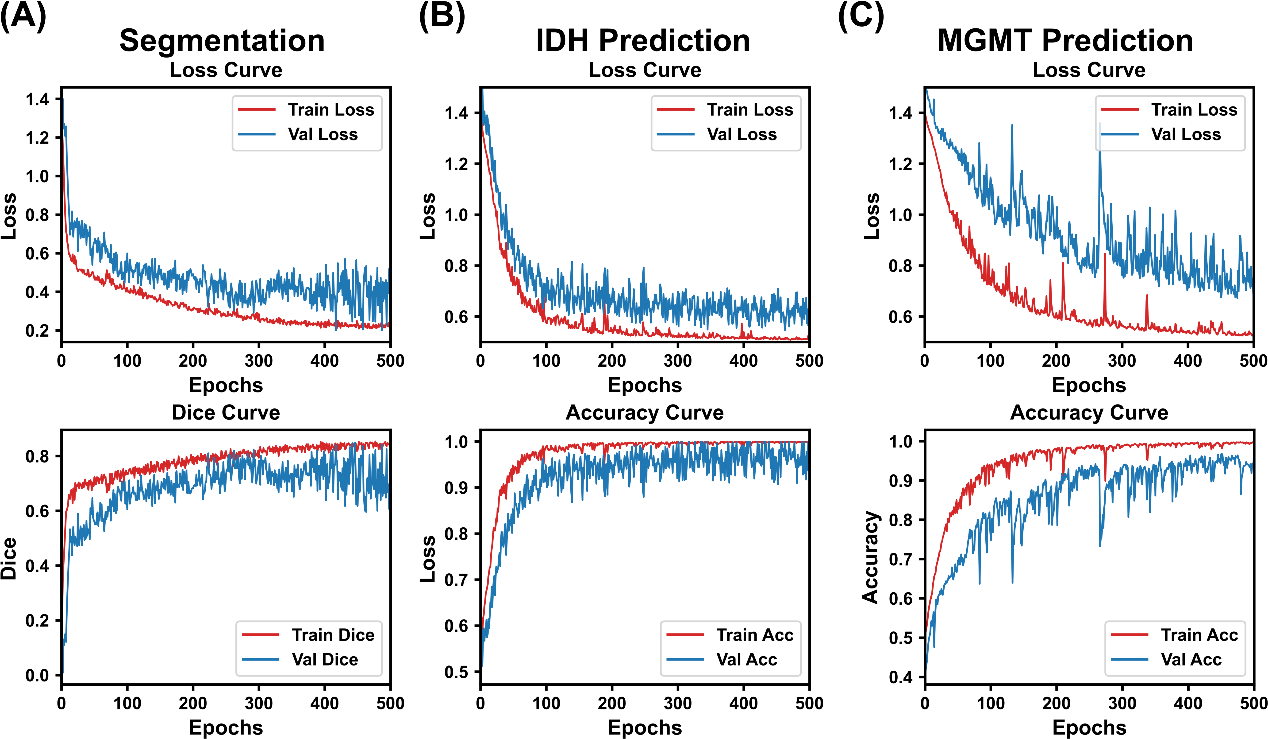


**Supplementary Figure S3.** Learning curves of different tasks. The training curves of the **(A)** segmentation, **(B)** IDH genotype prediction, and **(C)** MGMT genotype prediction with MixBranchNet. The red and blue lines are the curves of the training and validation sets, respectively.

## Network-learning evaluation

Five-fold cross-validation was conducted to evaluate network generalization. For each fold, the network was trained on 80% of the development set and evaluated on the held-out validation fold to obtain the corresponding metric (Dice coefficient for segmentation, accuracy for genotype prediction). Additionally, the coefficient of variation (CoV) was calculated to quantify the degree of fluctuation between folds, which was defined by:

(S3)

$\Delta(test-CV)=\text{Metric}_{test}-\overline{\text{Metric}}_{_{five-fold}}$ (S4)

**Table S6** summarizes the quantitative performance of each task from five-fold cross-validation. For all tasks, the CoV was low (Segmentation: 5.21%; IDH: 1.57%; MGMT: 3.45%), indicating limited variation in performance across folds and robustness to data partitioning. Furthermore, test performance had a similar range to the cross-validation performance (Segmentation, Δ(Test – CV) = 2.82; IDH, Δ(Test – CV) = -0.90; MGMT, Δ(Test – CV) = 0.11).

**Supplementary Table S6.** The performance from five-fold cross-validation and an independent test set.

| **Evaluation Metrics** | **CV (%)** | **CoV**  **(%)** | **95% CIs**  **(%)** | **Test**  **(%)** | **95% CIs**  **(%)** | **Δ (Test–CV)**  **(%)** | ***p*** |
| --- | --- | --- | --- | --- | --- | --- | --- |
| Dice  (Segmentation) | 81.27±3.52 | 5.21 | 76.90 – 85.64 | 84.09 | 79.62 – 88.52 | 2.82 | 0.10 |
| ACC  (IDH) | 95.90±1.51 | 1.57 | 94.21 – 97.68 | 95.00 | 93.72 – 96.13 | -0.90 | 0.07 |
| ACC  (MGMT) | 93.00±3.21 | 3.45 | 89.77 – 96.14 | 93.11 | 89.66 – 96.21 | 0.11 | 0.08 |

*p* values were calculated using Wilcoxon signed-rank tests applied to 2,000 paired bootstrap resamples of the cross-validation and test set. The five-fold cross-validation results are presented as mean ± standard deviation. CV = cross-validation; SD = standard deviation; CoV = coefficient of variation; CIs = confidence intervals; Dice = dice coefficient; ACC = accuracy.

# Section 5. CEST-Based Images of Different Contrasts for Segmentation.

To further investigate the impact of CEST on segmentation performance, 41 images were generated by varying the overall contrast of the normalized M_0_ image, corresponding to the 41 frequency offsets of the CEST image. The images were defined by:

$image_{i}=\alpha M_{_{0}}$ (S5)

Where α is contrast decay factor. We adjusted the overall contrast of M_0_ by setting α.

Ultimately, we obtained the M_0_ image (“Only M_0_ image”), images with copies of M_0_ (“Copy”), images with overall linear changes in M_0_ contrast (“Linear”), images whose M_0_ contrast follows the mean Z-spectrum within the brain (“Average”), and CEST images ranging from −5.0 to +5.0 ppm (“Full Z-spectra”). The representative visualized images of “Average” and “Full Z-spectra” are shown in **Figure S4**.

The segmentation results for the five input modalities are detailed in **Table S7**.


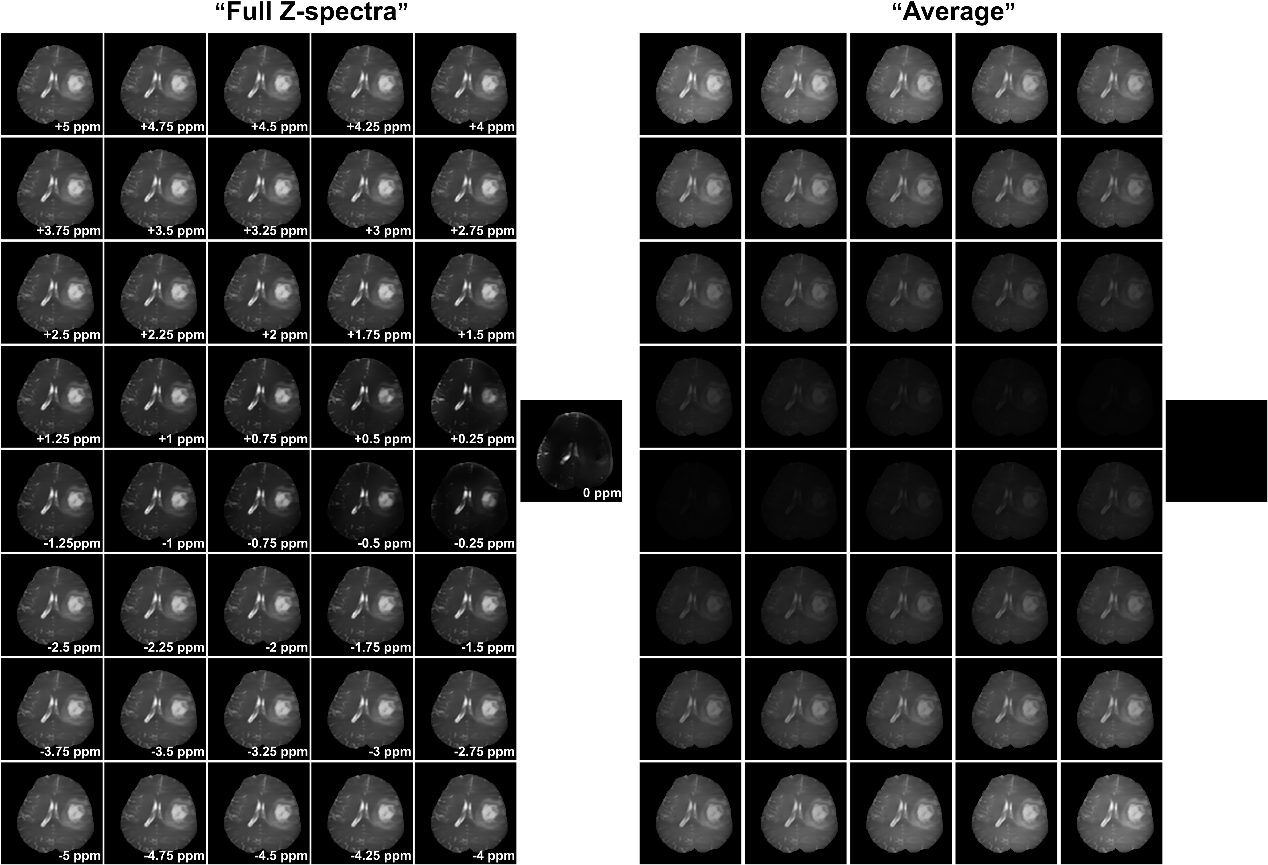


**Supplementary Figure S4.** Visualized input images of a glioma patient in the "Average" and "Full Z-spectra" modes. Images are arranged in a top-to-bottom, left-to-right order, with the central image listed separately on the far right.

**Supplementary Table S7.** Segmentation performance using different input modalities.

| **Input** | | **Dice** | **IoU** | **Accuracy** |
| --- | --- | --- | --- | --- |
| M_0_ image | Only M_0_ image | 75.97±1.14 | 63.66±1.62 | 96.20±0.35 |
|  | Copy | 75.97±1.14 | 63.66±1.62 | 96.20±0.35 |
|  | Linear | 75.95±1.11 | 63.64±1.61 | 96.16±0.35 |
|  | Average | 77.24±3.72 | 64.84±4.83 | 96.35±0.64 |
| Full Z-spectra | | **84.09±1.40** | **74.16±1.74** | **97.82±0.20** |

Comparison of segmentation performance across various input modalities using cross-validation. All metrics are reported as mean ± SD (%). Copy = input images with copies of M_0_ image; Linear = input images with overall linear changes in M_0_ contrast; Average = input images based on the overall M_0_ contrast varying with the mean Z-spectrum; Dice = dice coefficient; IoU = intersection over union.

# Section 6. Consistency analysis between patch-level and patient-level metrics.

To confirm that patch-based learning does not introduce optimistic bias, we performed an additional patient-level consistency analysis. **Figure S5** compares the patient-level and patch-level prediction probabilities (P_mean_). Across both tasks, the patch-level P_mean_ closely matched the corresponding patient-level P_mean_, with largely overlapping confidence intervals (IDH: 0.90–0.95 vs. 0.91–0.96; MGMT: 0.89–0.93 vs. 0.89–0.92). As shown in **Table S8**, no significant distributional differences were observed between the two levels (IDH: p = 0.209; MGMT: p = 0.197). Under the predefined equivalence margin of ±0.05, the two one-sided tests confirmed statistical equivalence in both tasks (IDH: mean difference = -0.0070, 90% CIs = -0.04 – 0.03, p = 0.019; MGMT: mean difference = 0.0013, 90% CIs = -0.02 – 0.03, p = 0.002). Overall, these findings indicate that patch-level evaluation produced results highly comparable to patient-level evaluation, with no evidence of optimistic bias.


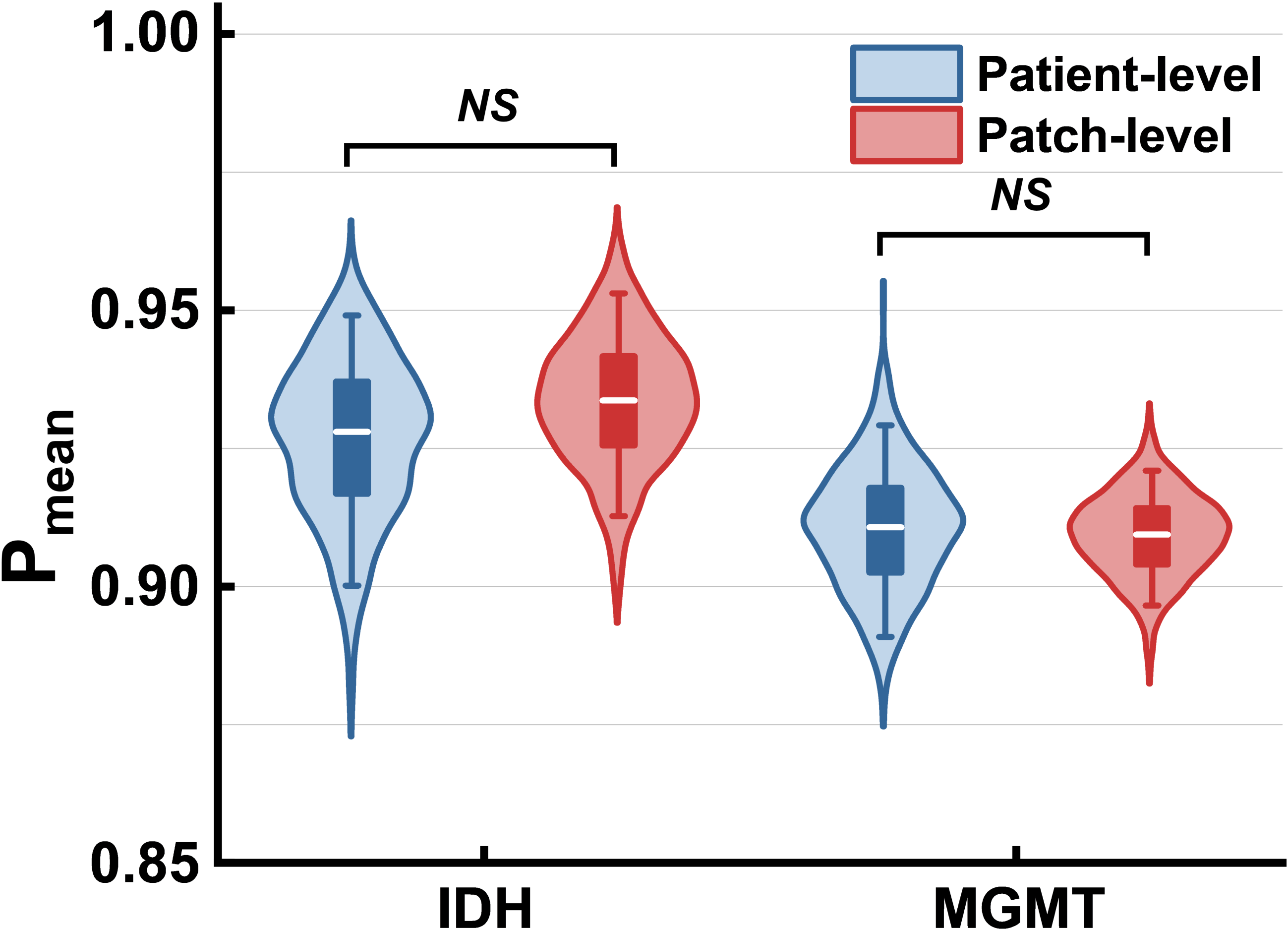


**Figure S5.** Comparability analysis between patient-level and patch-level prediction probabilities (P_mean_) for IDH and MGMT prediction. Violin plots illustrate the bootstrap distributions obtained from 2,000 resamples. Boxplots summarize the distributions of the performance metrics, with error bars indicating the 95% confidence intervals. The lower and upper edges of the box represent the first and third quartiles, respectively, and the white line indicates the median.

**Table S8.** Consistency analysis between patch-level and patient-level performance metrics.

| **Task (%)** | **Patch-P_mean_**  **[95% CIs]** | **Patient-P_mean_**  **[95% CIs]** | **ΔP_mean_**  **[90% CIs]** | **M–Whitney *p*** | **TOST *p*** |
| --- | --- | --- | --- | --- | --- |
| IDH Prediction | 93.36  [90.89, 95.82] | 92.66  [89.44, 95.27] | 0.70  [-2.34, 3.95] | 0.209 | 0.019 |
| MGMT Prediction | 90.94  [89.40, 92.32] | 91.07  [88.98, 93.37] | -0.13  [-2.44, 2.17] | 0.197 | 0.002 |

Values are presented as means with 95% confidence intervals (CIs), and the between-group difference (Δ) is reported with its 90% CIs. Confidence intervals were estimated using bootstrap resampling (B = 2000). Distributional differences were assessed using the Mann–Whitney U test on the original independent samples. Statistical equivalence was further evaluated using the two one-sided tests (TOST) procedure with a prespecified equivalence margin of ±0.05.

# Section 7. Small-sample robustness analysis for genotype prediction

To clarify the statistical uncertainty associated with subgroup size in genotype prediction, a supplementary patient-level robustness analysis was performed on the independent hold-out test set. The comparison was restricted to MixBranchNet and the U-Net baseline, because U-Net achieved the best performance among all baseline methods.

For each genotype task, patient-level paired differences in mean prediction probability (ΔP_mean_) were calculated, and the corresponding 95% confidence intervals (CIs) were estimated by paired bootstrap resampling (B = 2000). Statistical significance was assessed using a two-sided paired Wilcoxon signed-rank test. The two-sided exact paired sign-flip permutation test was used as an additional distribution-free robustness check to examine whether the observed superiority of MixBranchNet could plausibly arise by chance under random reassignment of the paired difference signs. In addition, power was estimated using a simulation-based paired Wilcoxon framework at the observed hold-out sample size. Estimated power was defined as the proportion of simulated paired datasets in which the superiority of MixBranchNet over U-Net remained statistically significant. The simulated paired datasets were generated by ΔP_mean_ and standard deviation (SD) of the paired differences. Following conventional practice, a power of 80% was considered acceptable for statistical detectability. A threshold of *p* ≤ 0.05 was considered statistically significant.

As shown in **Table S9**, Wilcoxon testing indicated significantly higher P_mean_ in MixBranchNet for both IDH (*p* = 0.006) and MGMT (*p* = 0.039). The permutation results were consistent (IDH: *p* < 0.001; MGMT: *p* = 0.008), further supporting that the observed advantage was unlikely to be attributable solely to a favorable configuration of a small number of paired observations. The estimated power was 95.7% for IDH and 88.4% for MGMT, indicating that the observed advantage of MixBranchNet over U-Net was statistically detectable under the current sample size, although the smaller MGMT subgroup still warrants cautious interpretation regarding the stability of performance estimation.

**Table S9.** Small-sample robustness analysis for genotype prediction on the hold-out test set.

| **Genotype** | **Hold-out patients (n)** | **ΔP_mean_**  **(%)** | **SD**  **(%)** | **95% CIs**  **(%)** | **Wilcoxon**  ***p*** | **Permutation *p*** | **Power**  **(%)** |
| --- | --- | --- | --- | --- | --- | --- | --- |
| **IDH** | 14 | 3.62 | 3.40 | 2.45 – 4.83 | 0.006 | <0.001 | 95.7 |
| **MGMT** | 8 | 4.50 | 3.43 | 2.51 – 6.89 | 0.039 | 0.008 | 88.4 |

Values are reported at the patient level on the hold-out test set for the comparison between MixBranchNet and U-Net. ΔP_mean_ indicates the mean paired difference in prediction probability, and SD represents the standard deviation of paired differences. The 95% confidence intervals (CIs) for ΔP_mean_ were estimated by paired bootstrap resampling (B = 2,000). Wilcoxon *p* values were obtained using two-sided paired Wilcoxon signed-rank tests. Permutation *p* values were obtained using two-sided exact paired sign-flip permutation tests based on the observed patient-level paired differences. Power was estimated using a simulation-based paired Wilcoxon framework at the observed hold-out sample size.

# Section 8. Impact of B_0_ Correction on Model Performance

To assess whether our MixBranchNet could intrinsically handle the B_0_ correction issue while performing predictions, we performed a comparative analysis between CEST data that underwent B_0_ correction and uncorrected CEST data in the hold-out test set. In our dataset, the measured B_0_ field maps showed offsets on the order of ± 0.3 ppm, consistent with expected field inhomogeneities at the given field strength and shim conditions. The corrected data underwent standard B_0_ inhomogeneity correction as described in the Materials and Methods section.

As shown in **Figure S6**, there was a slight reduction in Dice score when using uncorrected data compared to corrected data, but the difference was not statistically significant (*p* = 0.210). For both IDH and MGMT genotype prediction tasks, the F1-scores were comparable between the corrected and uncorrected data (within a 1-2% difference; IDH: *p* = 0.163; MGMT: *p* = 0.351). Additionally, given that some CEST data in our dataset exhibited more pronounced B_0_ inhomogeneities (about ± 1.0 ppm), we conducted further experiments by applying offset shifts to the Z-spectrum to simulate larger B_0_ distortions within the test set. All performance metrics exhibited significant decreases (*p* < 0.001).


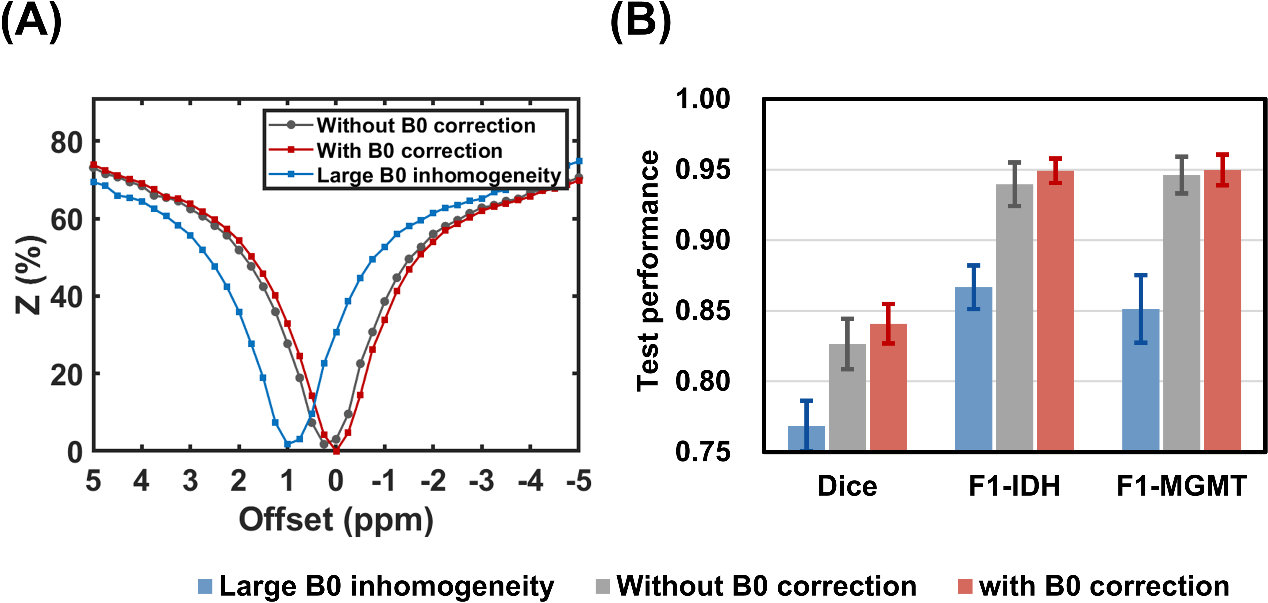


**Supplementary Figure S6.** Impact of B_0_ correction on model performance for CEST data analysis. **(A)** Representative Z-spectra taken from glioma regions, showing the effects of different CEST data preprocessing: without B_0_ correction (gray), B_0_ correction (red), and large B_0_ inhomogeneity (blue). **(B)** Performance metrics using three inputs, including the Dice score of segmentation (Dice), the F1-score of IDH prediction (F1-IDH), and the F1-score of MGMT prediction (F1-MGMT). Results are presented as mean ± standard deviation from hold-out test set.

# Section 9. Noise-injection robustness test.

To evaluate the robustness of MixBranchNet to degraded image quality, we performed noise injection on the independent hold-out test set. For each patient, we simulated MRI magnitude noise using a Rician model by adding Gaussian noise to the real and imaginary components of both the multi-offset CEST images (41 offsets) and the reference image M_0_​ image, followed by a magnitude operation:

$\tilde{S}=\sqrt{(S+n_{r})^{2}+{n_{i}}^{2}},\text{ }n_{r},n_{i}\sim N(0, \sigma^{2})$ (S6)

Where $S$ is CEST image with a single offset. $n_{r}$ and $n_{i}$ denote independent zero-mean Gaussian noise terms added to the real and imaginary components. Noise levels were parameterized by target $SNR$ values {40, 30, 20, 10, 5}, where $\sigma=A/SNR$ and $A$ was defined as the median magnitude of M_0​_ within the tumor mask for each patient. For each noise level, inference was repeated across five random seeds to reduce stochastic variability. The CEST images were normalized after adding noise.

As shown in **Figure S7**, Accuracy showed the expected monotonic decrease with increasing noise. Relative to the clean condition (IDH: 95.00%; MGMT: 93.11%), performance was largely preserved under mild perturbations (SNR ≥ 30; IDH: 93.79% - 95.00%; MGMT: 90.79% - 93.11%), whereas a larger drop was observed under more severe noise (SNR ≤ 30; IDH: 78.08% - 93.79%; MGMT: 70.89% - 90.79%). The standard deviation widened at higher noise, consistent with increased prediction uncertainty as acquisition quality degraded.


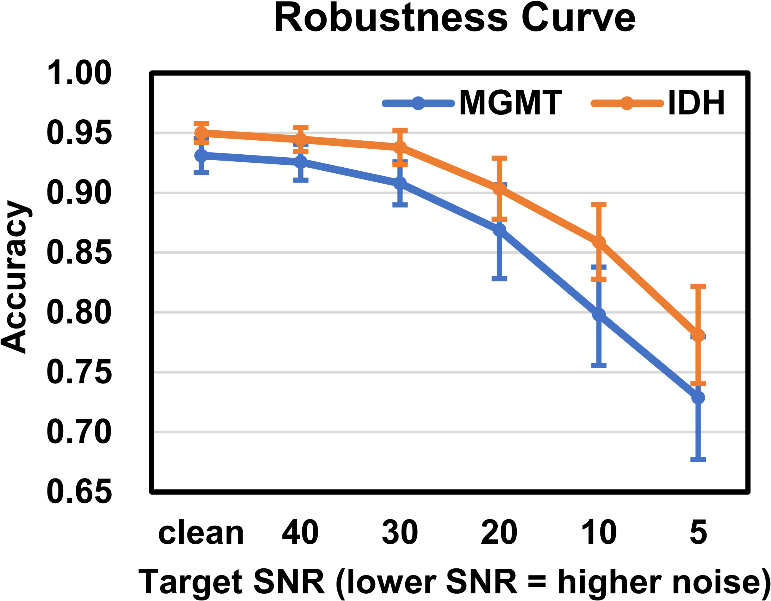


**Supplementary Figure S7.** Robustness to acquisition-quality degradation via Rician noise injection. Test-time Rician noise was injected into both multi-offset CEST images and M_0_, followed by re-computation of the normalization input. Curves show accuracy as a function of target signal-to-noise ratio (SNR). Error bars represent the standard deviation across folds.
